# Dissecting the role of crosstalk between glioblastoma subpopulations in tumor cell spreading

**DOI:** 10.1038/s41389-020-0199-y

**Published:** 2020-02-05

**Authors:** Maria R. Jubran, Ariel M. Rubinstein, Irina Cojocari, Ibukun Adesoji Adejumobi, Maxim Mogilevsky, Sama Tibi, Ronit V. Sionov, Maïté Verreault, Ahmed Idbaih, Rotem Karni, Nataly Kravchenko-Balasha

**Affiliations:** 1 Department for Bio-Medical Research, Faculty of Dental Medicine, Hebrew University of Jerusalem, Jerusalem, 91120 Israel; 2 Department of Biochemistry and Molecular Biology, Institute for Medical Research Israel-Canada, Hebrew University-Hadassah Medical School, Jerusalem, 9112001 Israel; 3 Sorbonne Université, Inserm, CNRS, UMR S 1127, Institut du Cerveau et de la Moelle épinière, ICM, AP-HP, Hôpitaux Universitaires La Pitié Salpêtrière, Service de Neurologie 2-Mazarin, F-75013 Paris, France

**Keywords:** Cell migration, Tumour heterogeneity

## Abstract

Glioblastoma (GBM) is a highly infiltrative brain cancer, which is thus difficult to operate. GBM cells frequently harbor Epidermal Growth Factor Receptor amplification (EGFRwt) and/or activating mutation (EGFRvIII), generating at least two different cellular subpopulations within the tumor. We examined the relationship between the diffusive architectures of GBM tumors and the paracrine interactions between those subpopulations. Our aim was to shed light on what drives GBM cells to reach large cell–cell distances, and whether this characteristic can be manipulated. We established a methodology that quantifies the infiltration abilities of cancer cells through computation of cell–cell separation distance distributions in 3D. We found that aggressive EGFRvIII cells modulate the migration and infiltrative properties of EGFRwt cells. EGFRvIII cells secrete HGF and IL6, leading to enhanced activity of Src protein in EGFRwt cells, and rendering EGFRwt cells higher velocity and augmented ability to spread. Src inhibitor, dasatinib, at low non-toxic concentrations, reduced the infiltrative properties of EGFRvIII/EGFRwt neurospheres. Furthermore, dasatinib treatment induced compact multicellular microstructure packing of EGFRvIII/EGFRwt cells, impairing their ability to spread. Prevention of cellular infiltration or induction of compact microstructures may assist the detection of GBM tumors and tumor remnants in the brains and improve their surgical removal.

## Introduction

GBM is an aggressive and highly infiltrative brain cancer that is highly tolerant to anticancer drugs^1,2^. The current standard of care for GBM tumors is surgery, followed by radiation treatment with concurrent and adjuvant temozolomide chemotherapy^3^, with median survival rates of 15 months^4^. The majority of GBM tumors relapse, with a median recurrence period of 7 months^4^. One of the main reasons for poor patient survival is the diffusive nature of these tumors^5^, resulting in incomplete microscopic resection of the tumors, and failure of the concurrent radiotherapy and chemotherapy to control the cancer burden. Indeed, several studies demonstrated that the recurrent tumors appear within 2 cm of the primary tumor site in 90–95% of cases, suggesting that invading cells are poorly affected by the available treatments^6,7^. Thus, in order to help increase successful therapeutic outcomes a strategy addressing the infiltrative nature of the tumor is highly demanded.

To propose such a strategy, we investigated the relationship between the infiltrative nature of GBM tumor cells and the mechanism of interaction between two main GBM cellular subtypes: a cellular subtype harboring mutation in epidermal growth factor receptor (EGFR), EGFRvIII, and a subtype overexpressing wild type EGFR (EGFRwt). EGFRvIII mutation gives rise to constitutive activation of EGFR receptor and its downstream signaling^8,9^. Although EGFRvIII cells comprise only a minor subpopulation in GBM, the EGFRvIII subtype was found to be responsible for enhanced proliferation of certain GBM tumors^9^. We have recently demonstrated that the diffusive nature of EGFRvIII cells can be mediated by cell–cell homogeneous interactions within the EGFRvIII subpopulation^10^. In the current study we took a step forward by examining the relationship between the diffusive architectures of GBM tumors harboring EGFRvIII mutation and paracrine, heterogeneous interactions between EGFRwt and EGFRvIII cellular subtypes.

Utilizing U87EGFRwt and U87EGFRvIII cell lines as representatives of EGFRwt and EGFRvIII cellular subpopulations in GBM tumors^9^, respectively, we found that the aggressive EGFRvIII cells modulate the migration and infiltrative properties of EGFRwt cells through Src activation. Activated Src was associated with enhanced cellular velocity, induced length of movement trajectories and improved ability of EGFRwt cells to spread in 2D and 3D cellular cultures. Low, non-toxic concentrations of dasatinib, a Src inhibitor, significantly affected the migration and diffusive properties of EGFRwt cells induced by EGFRvIII cells, as well as of GBM cellular populations comprised of both EGFRwt and EGFRvIII subpopulations. HGF and IL6 ligands, secreted by EGFRvIII cells, were required to maintain the enhanced activity of Src protein in EGFRwt cells.

In summary, we show that EGFRvIII-driven crosstalk between EGFRvIII and EGFRwt subtypes enhances the diffusive properties of the brain tumor cellular population. We show that scattered distributions of GBM populations possessing EGFRvIII subpopulations can be controlled by Src inhibition. We validate the potential clinical relevance of this result by using patient-derived GBM cells harboring EGFRvIII mutation. As the cancer clinics move towards personalized treatments, and once EGFRvIII mutation can be detected in cerebrospinal fluid through liquid biopsy^11^, non-toxic Src inhibition can be further explored as a strategy to enhance the success of surgical removal of GBM tumors harboring EGFRvIII mutation.

## Results

### EGFRvIII cells exhibit a scattered distribution in 3D extracellular matrix (ECM), whereas EGFRwt are cells closely packed

It has been shown that a small subpopulation of EGFRvIII cells within the tumor is responsible for GBM proliferation through cell–cell communication^9^. We wished to study how these interactions affect the ability of GBM cells to spread.

**Fig. 1:**
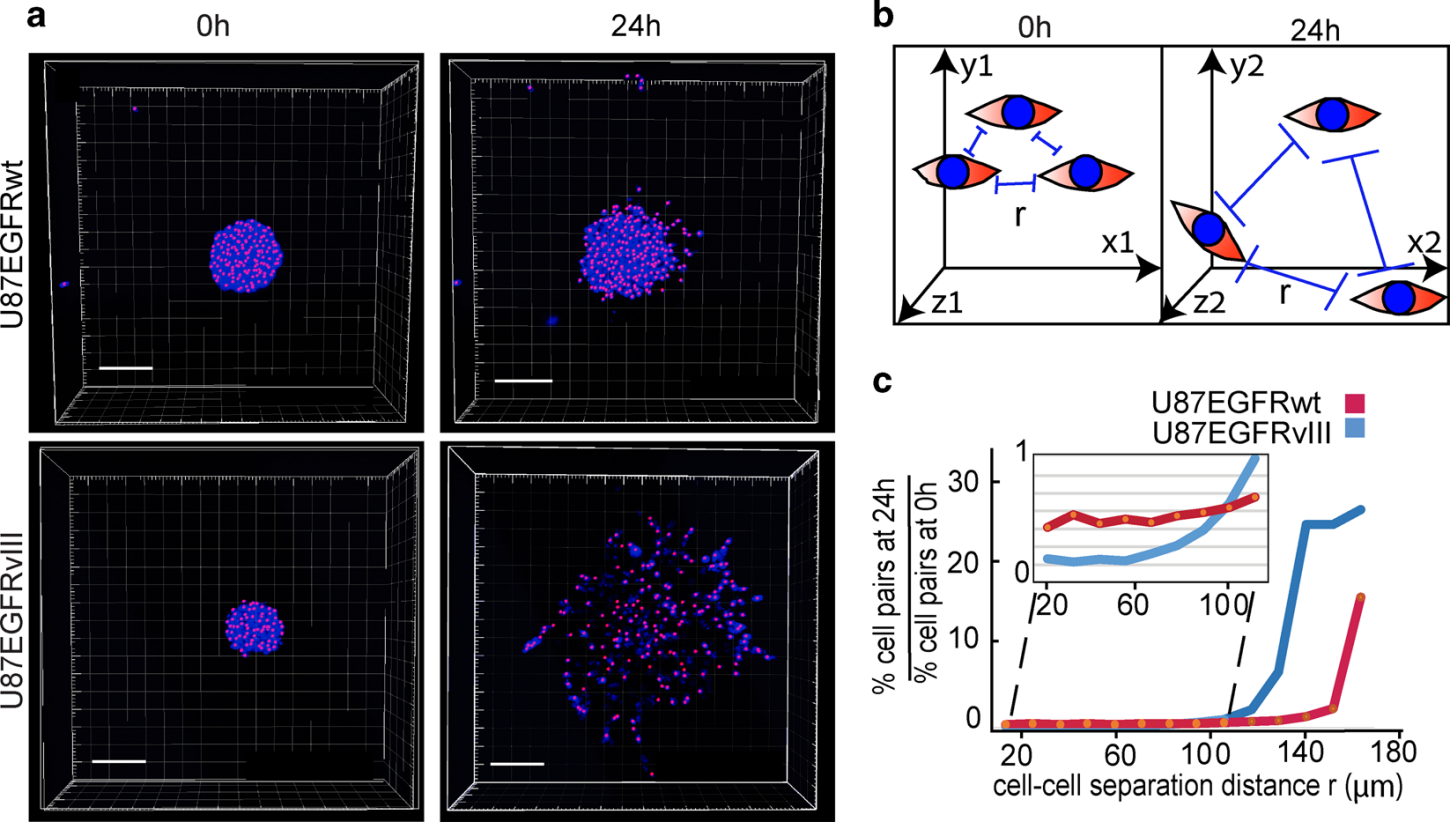
U87EGFRvIII neurospheres demonstrate enhanced infiltrative properties in comparison with U87EGFRwt neurospheres. **a** GBM neurospheres (NS) were embedded into 40% Matrigel (U87EGFRwt NS are shown in upper panel, and U87EGFRvIII NS in lower panel). Cell nuclei were imaged at 0 h (left panel) and 24 h (right panels) using confocal microscopy. Pink dots represent geometric centers of each nuclei which were used to define the cell coordinates. These coordinates were used to calculate cell–cell distances as described in Methods. Scale bars represent 150 μm. **b** Cell–cell separation distance (*r*) was calculated as described in Methods. All cell pairs, up to a separation distance of 200 μm were measured. **c** Fold change in the percentage of the cell pairs located at certain cell–cell distance, *r*, after 24 h relative to 0 h is presented in the plot. At least eight neurospheres were analyzed for each condition.

First, we examined the infiltration and migration properties of each GBM subtype. For this purpose we utilized U87EGFRvIII and U87EGFRwt cells as representatives of GBM EGFRvIII and EGFRwt subtypes^9^, respectively, and examined their properties in a 3D environment. The importance of the 3D environment in oncogenic processes, and specifically in tumor cell migration is well documented^12,13^. We constructed an experimental-computational 3D model allowing to detect cell–cell locations in matrigel through identification of *X*, *Y*, and *Z* coordinates and then utilized them to quantify the spreading abilities of the cells by calculating distributions of cell–cell separation distances (Methods). Figure 1a clearly shows that U87EGFRvIII neurospheres spread out to longer distances than U87EGFRwt neurospheres, indicating that similar phenotype characteristics, as seen in 2D^10^, were preserved in the 3D ECM models. Quantification of cell–cell separation distances (Fig. 1b, c) supported these results, showing that a higher percentage of U87EGFRwt cell pairs were separated by shorter distances (<100 μm, red curve in the inset plot of Fig. 1c) than the percentage of U87EGFRvIII cell pairs after 24 h (blue curve, Fig. 1c). Many more U87EGFRvIII cell pairs were found at large (>100 μm) cell–cell separation distances after 24 h in comparison with U87EGFRwt cell pairs (Fig. 1c).

Moreover, we monitored cell–cell distances immediately following cell division, and found that the daughter U87EGFRvIII cells moved away from each other faster and reached larger cell–cell separation distances, relative to U87EGFRwt cells (Video 1A,B and Fig. S1A).

These results indicate that U87EGFRvIII cells, in addition to their role as drivers of GBM tumor proliferation^9^, also possess an increased ability to migrate and infiltrate, which renders them to be potentially more aggressive.

### EGFRvIII cells enhance the migration and infiltration properties of EGFRwt cells

**Fig. 2:**
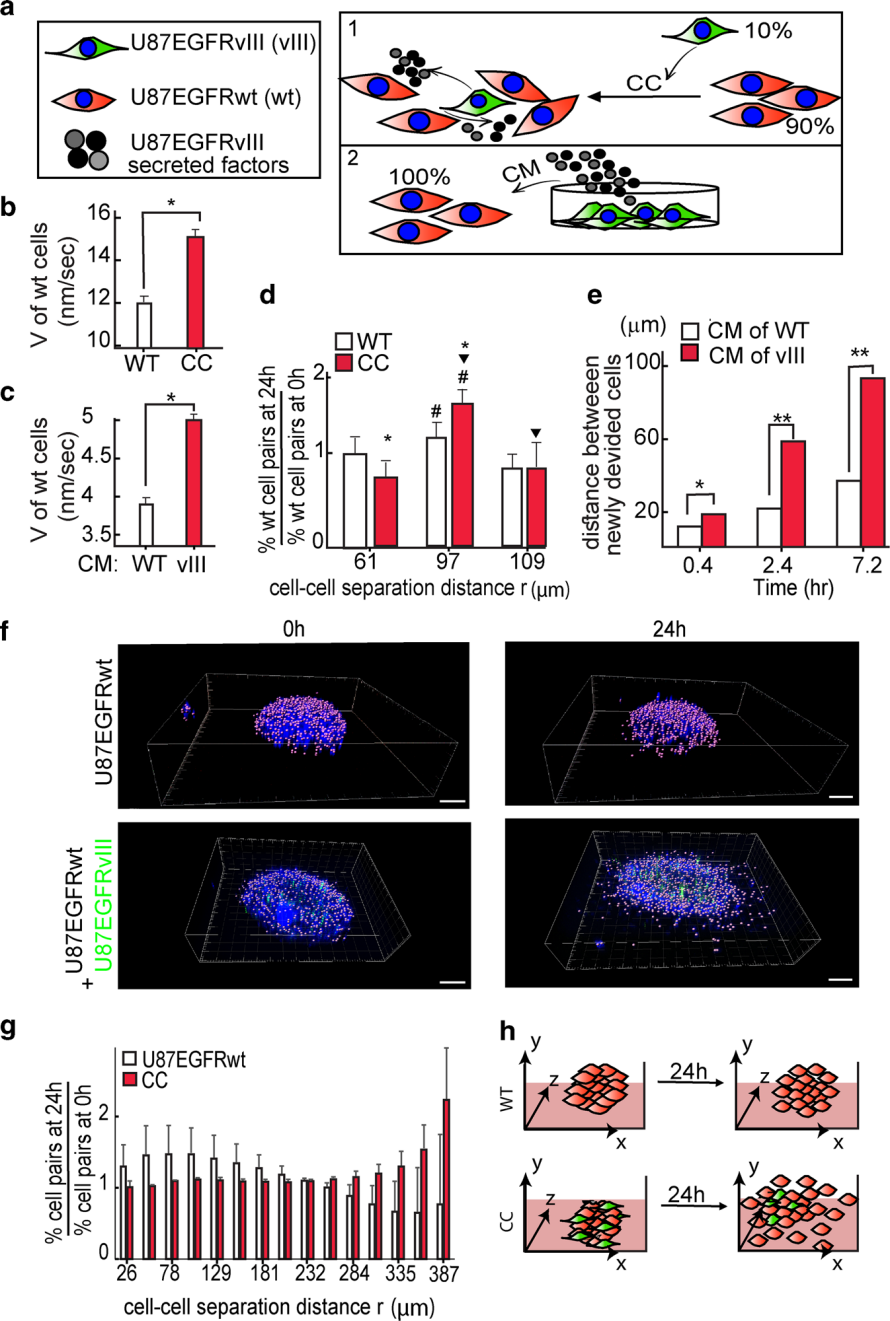
U87EGFRvIII cells modulate the migration and spreading properties of U87EGFRwt cells. **a** Effect of U87EGFRvIII cells (shown in green) on U87EGFRwt cells (shown in red) was examined by either co-culture (CC) or by stimulating U87EGFRwt cells with conditioned medium (CM) of U87EGFRvIII as shown in the illustration. **b**, **c** Change in the cell velocity (*V*) is shown for U87EGFRwt cells which were cultured for 24 h with either 10% of U87EGFRvIII cells (*P *<* 0.001) (**b**) or under CM of U87EGFRvIII cells (CM (vIII), *P *<* 0.001) (**c**). **d** U87EGFRwt cells were either cultured with 10% of U87EGFRvIII or alone. The fold change in the percentage of the cell pairs located at certain cell–cell distance, *r*, after 24 h relative to 0 h was calculated (the symbols #, ▾ indicate *P* *<* 0.05, the symbol * indicates *P* *<* 0.01). **e** U87EGFRwt cells were incubated with either U87EGFRvIII CM (vIII) or their own CM (WT) medium. U87EGFRwt dividing cells were imaged using live-cell NIS-Elements—Microscope Imaging. Cell–cell distances of dividing cells were measured every 20 min. Three representative time intervals are shown; *n* = 12 cell pairs were used for each condition (*P *<* 0.05; **P *<* 0.001). **f** U87EGFRwt neurospheres are shown in the upper panel and CC in the lower panel. Hoechst-labeled cell nuclei were imaged at 0 h (left panel) and 24 h (right panels) using confocal microscopy. U87EGFRwt cells were labeled with Qtracker 705, shown in green in the figure. Scale bars represent 100 μm. **g** The fold change in cell–cell separation distances were calculated after 24 h relative to 0 h. **h** The results in (**a**–**g**) suggest that U87EGFRvIII cells modulate the migration and spreading properties of U87EGFRwt through soluble factors secreted from U87EGFRvIII cells.

We hypothesized that the infiltrative architectures of GBM tumors might be maintained by the heterogeneous interactions between the aggressive EGFRvIII cells and the less aggressive EGFRwt cells within the tumor. To examine this hypothesis, we measured the migration and infiltration properties of U87EGFRwt cells when they were either co-cultured (CC) with U87EGFRvIII cells or were grown under medium that was conditioned by EGFRvIII cells, in 2D cultures (conditioned medium, CM; Fig. 2a). Figure 2 demonstrates that U87EGFRvIII cells increased the velocity of U87EGFRwt cells when U87EGFRwt cells were co-cultured with U87EGFRvIII cells (Fig. 2b) and also when they were grown under U87EGFRvIII CM (Fig. 2c). Furthermore, U87EGFRvIII cells increased the ability of the less aggressive U87EGFRwt cells to spread out in co-culture (Fig. 2d). Time course monitoring of U87EGFRwt cells, immediately following cell division, revealed that daughter U87EGFRwt cells spread out much faster when they were cultured in CM of EGFRvIII cells relative to U87EGFRwt condition medium, demonstrating a stronger cell–cell repulsion induced by U87EGFRvIII (Fig. 2e). These results suggest that U87EGFRvIII cells can modulate the migration and infiltration properties of U87EGFRwt cells in 2D cultures.

To verify that the U87EGFRvIII/U87EGFRwt crosstalk was not limited to 2D cultures, we generated neurospheres comprising U87EGFRvIII and U87EGFRwt cells, and embedded them in ECM. U87EGFRvIII cells constituted 10% of the entire cellular population (Fig. 2f). The co-culture of U87EGFRvIII/U87EGFRwt induced the spreading abilities of U87EGFRwt cells after 24 h (Fig. 2f, lower panel). Calculation of cell–cell separation distances revealed that U87EGFRwt cells reached longer cell–cell separation distances after 24 h in comparison with homogeneous U87EGFRwt neurospheres (Fig. 2g, h).

**Fig. 3:**
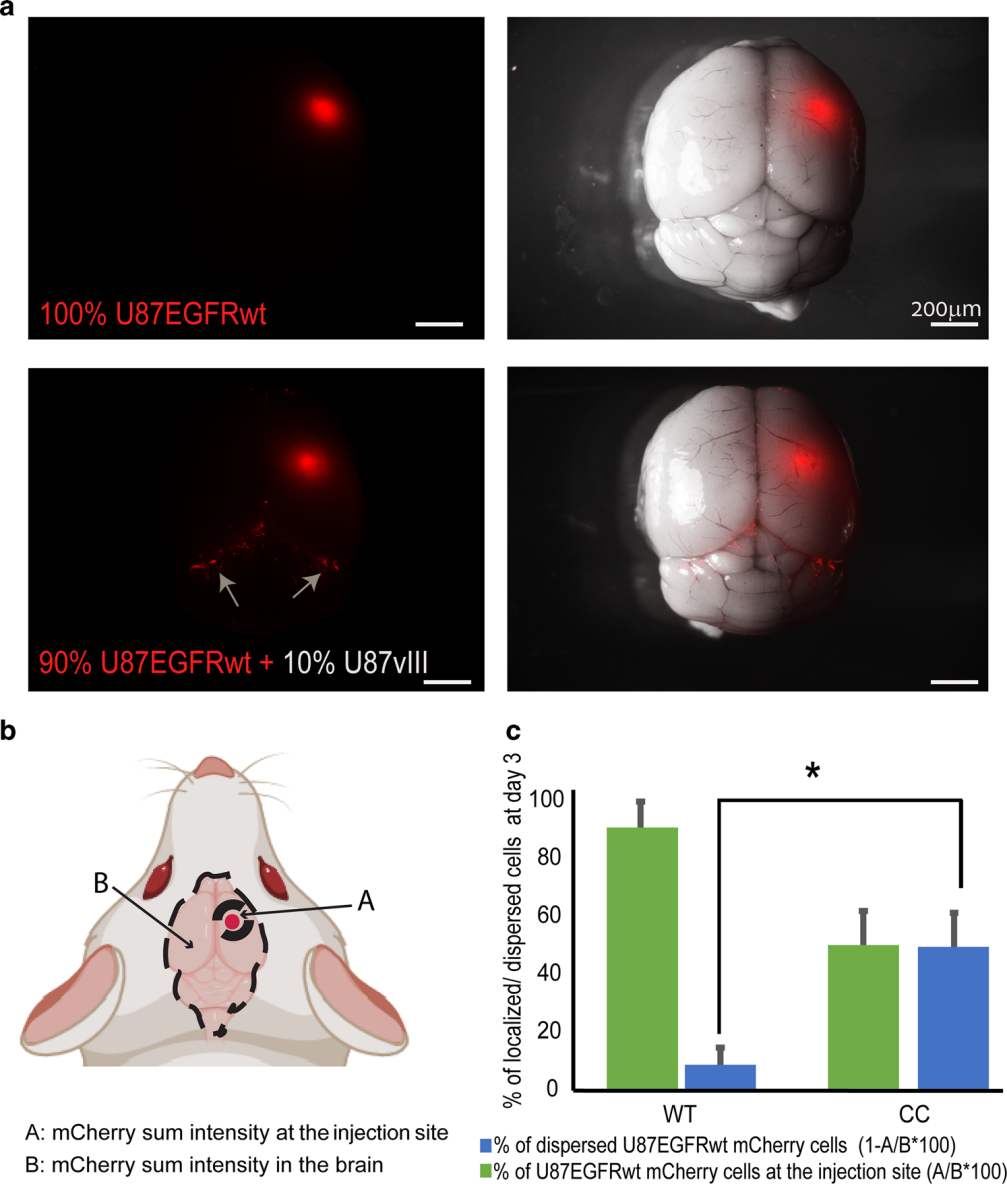
Mixed tumors (10% U87EGFRvIII, 90% U87EGFRwt) show increased infiltrative properties in vivo. **a** mCherry-labeled U87EGFRwt cells were co-injected intracranially with unlabeled U87EGFRvIII cells (U87vIII), in ratio of 9:1 (or only U87EGFRwt cells as control), into the NSG mice striatum. Three days later the mice were euthanized and brains were imaged under a fluorescent dissecting microscope. **b**, **c** Index of % of the cell dispersion was calculated (**c**) comparing the sum intensity from the primary tumor to the dispersed cells in the brain as indicated in (**b**). *P *=* *0.01*, *n* = 6 mice per each group. Values are standard error (SE).

Furthermore, examination of brain tumors generated in NSG mice from 10% of U87EGFRvIII cells and 90% of U87EGFRwt cells, revealed that the mixed U87EGFRvIII/U87EGFRwt tumors had higher infiltrative properties relative to tumors generated from 100% of U87EGFRwt cells (Fig. 3).

These results not only confirm the observations demonstrating that tumors harboring EGFRvIII mutation are more aggressive^9,14^, but also point to the ability of U87EGFRvIII cells to induce the infiltrative properties of U87EGFRwt cells in vivo.

### U87EGFRvIII cells induce Src activation in U87EGFRwt cells

To examine the molecular mechanism behind the crosstalk that mediates the induction of U87EGFRwt cell spreading, we examined proteins that could transmit the U87EGFRvIII/U87EGFRwt communication into the induced movement of U87EGFRwt cells.

U87EGFRwt cells were either co-cultured with U87EGFRvIII cells in a ratio of 9:1, or were grown in U87EGFRvIII CM. Induction of pSrc, pFAK and pmTOR was observed, when U87EGFRwt cells were co-cultured with U87EGFRvIII cells, as represented by induced phosphorylation of these proteins (Fig. S1B). A truncated form of EGFR could be detected in U87EGFRvIII cell cultures or U87EGFRvIII/U87EGFRwt co-cultures (Fig. S1B), corresponding to the fact that U87EGFRvIII cells have a deletion mutation in EGFR extracellular domain.

**Fig. 4:**
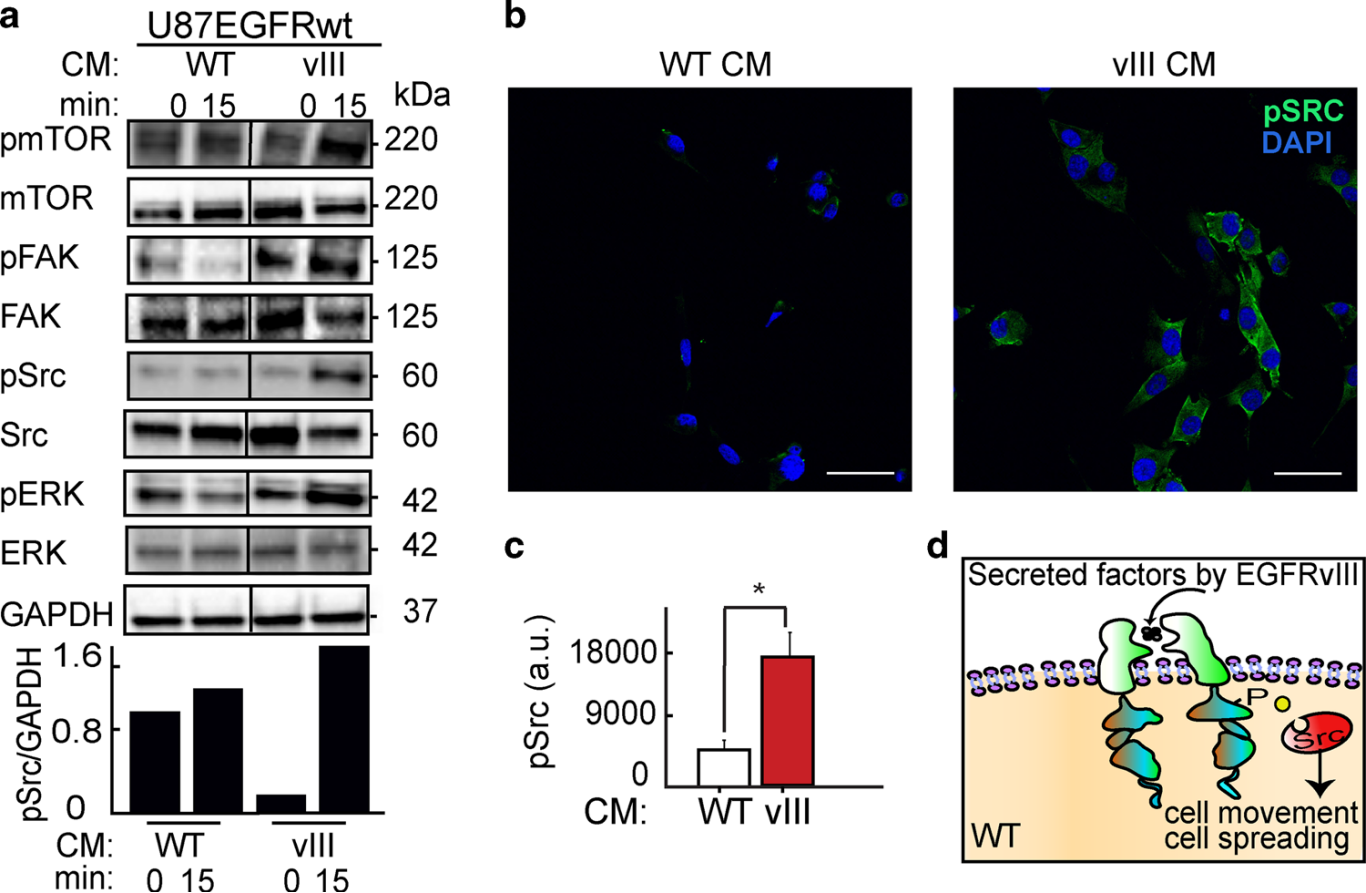
Src mediates U87EGFRvIII/ U87EGFRwt crosstalk. **a** Upper panel: Cells were grown under either U87EGFRwt conditioned medium (CM WT) or under U87EGFRvIII CM (CM vIII). Western blot assay was performed to examine the effect of U87EGFRvIII CM (vIII) on U87EGFRwt cells after 15 min. Bottom panel: quantification of pSrc levels normalized to GAPDH. All the proteins in the tested samples were assayed on the same membrane. The line in the middle indicates that non-relevant samples are not shown. **b** Representative images of immunofluorescence assays show Src activation (as represented by increase in pSrc) in U87EGFRwt cells following stimulation with CM of U87EGFRvIII (vIII). ×40 lens; scale bars represent 150 μm. **c** Average of pSrc intensity was quantified from eight fields (~20 cells/field). Plot is representative of at least three independent experiments, (*P *=* 0.005). Values are standard error (SE). **d** Schematic representation of Src activation in U87EGFRwt cells in response to EGFRVIII CM.

CM of U87EGFRvIII cells induced activation of Src, mTOR, and FAK proteins as well, as represented by induced phosphorylation levels of those proteins in U87EGFRwt cells (Fig. 4a).

Src activation in response to U87EGFRvIII CM was validated further using immunofluorescence staining. 3.5-fold Src activation was detected in U87EGFRwt cells in response to CM from U87EGFRvIII cells (Fig. 4b, c).

These results suggest that U87EGFRvIII cells activate Src protein in U87EGFRwt cells through secreted factors (Fig. 4d). Interestingly 10% of U87EGFRA289V cells (U87EGFRwt cells, which have been engineered to harbor activating EGFR mutation, A289V^15^) failed to increase pSrc levels (Fig. S2A,B) in U87EGFRwt cells. Furthermore U87EGFRA289V cells did not signifcantly induce the infiltrative properties of U87EGFRwt cells as shown in Fig. S2C. This result suggests that EGFRvIII mutation may provide a specific biomarker for the increased infiltration of GBM cells due to communication between the GBM cell populations.

### Src activation is required for induced infiltration and spreading of the cells in heterogeneous GBM population

**Fig. 5:**
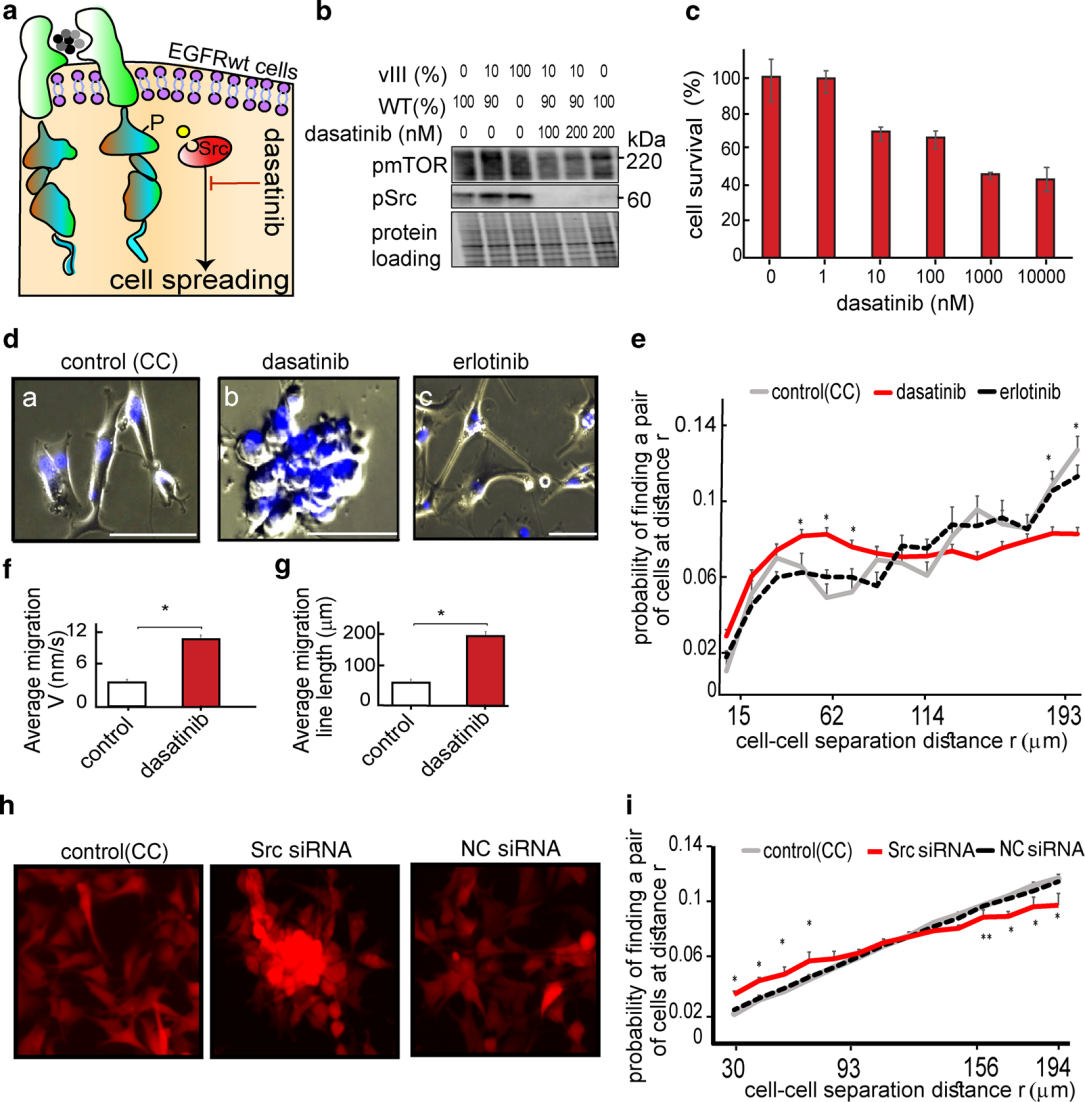
Dasatinib (Src inhibitor) inhibits spreading of EGFRvIII/EGFRwt multicellular cultures. **a** We hypothesize that pSrc inhibition, using, for example, dasatinib at non-toxic concentrations, may reduce the spreading of EGFRvIII/EGFRwt CC. **b** Western blot analysis shows that Src activity was inhibited using non-toxic concentration (100–200 nM) of dasatinib in CC after 24 h of treatment. WB images are representative of at least three independent experiments. **c** Examination of CC survival, treated with a range of dasatinib concentrations, for 72 h. Bars represent % of viable cells as assessed by Methylene blue assay (error bars represent SE). **d** CC was treated for 24 h with 100 nM of dasatinib or 100 nM erlotinib. Hoechst-labeled cell nuclei were imaged using live-cell imaging chambers (Eclipse Ti-E, Nikon inverted microscope Scale bars, 100 μm, ×10 lens). **e** Probability of finding a pair of the U87EGFRwt cells at r (0–200 μm) was calculated at 0 and 72 h as described in Methods. Plots represent the distribution of cell–cell separation distances in CC treated with dasatinib, with erlotinib or in the untreated CC. *P *≤* 0.03 was found for CC treated with dasatinib vs. untreated and for CC treated with dasatinib vs erlotinib. **f**, **g** CC (Control) and CC treated with dasatinib for 18 h were imaged using ×10 lens and live-cell imaging chambers. Cell velocity, *V* (**f**) and the line length of cell migration (**g**) were calculated using the NIS-Elements (Nikon); *P *<* 0.001. Data were collected from eight fields (~200 cells/field). See also video 2(A and B). **h**, **i** mCherry-labeled U87EGFRwt cells were transfected with 0.5 nM Src siRNA or negative control (NC) siRNA. Non-transfected (indicated as control) and siRNA transfected U87EGFRwt cells were co-cultured with U87EGFRvIII cells (CC), in ratio of 9:1. 72 h after seeding, cells were imaged, and the probability of finding a pair of the U87EGFRwt cells at *r* (0–200 μm) was calculated as described in Methods. Plots represent the distribution of cell–cell separation distances in untreated CC or CC transfected with 0.5 nM Src/NC siRNA. *P *<* 0.001, **P *=* 0.01 (for CC treated with Src siRNA vs untreated). Data were collected from eight fields (~200 cells/field).

Accumulating data demonstrate that Src kinase is a promising target for anticancer therapy^16^. However, clinical trials failed to demonstrate significant effects of anti-Src agents on GBM growth^17^. We hypothesized that although Src inhibitors might be ineffective in inhibition of GBM growth, they may be beneficial in stopping tumor spreading. This approach can be particularly effective in tumors harboring a U87EGFRvIII population. Thus, we assessed the effect of Src inhibition on the infiltrative properties of the heterogeneous GBM model. For this purpose we utilized dasatinib (Fig. 5a), an FDA-approved Src family inhibitor. Dasatinib, at concentrations of 100–200 nM, inhibited Src in U87EGFRwt cells that were co-cultured with 10% of U87EGFRvIII cells (Fig. 5b). At these concentrations most of the cells survived the 72 h dasatinib treatment (Fig. 5c), but their spreading ability was inhibited (Fig. 5d, subpanels a and b). Calculation of cell–cell separation distances supported these results, showing that dasatinib induced packing of U87EGFRwt cells, as higher number of U87EGFRwt cell pairs were found to be located at short cell–cell separation distances following dasatinib treatment (Fig. 5e). Moreover, dasatinib induced increased U87EGFRwt cell velocity and directed cell–cell movement towards each other (Fig. 5f, g), thereby forming multicellular clusters from single cells (Fig. 5d, Video 2A, B).

**Fig. 6:**
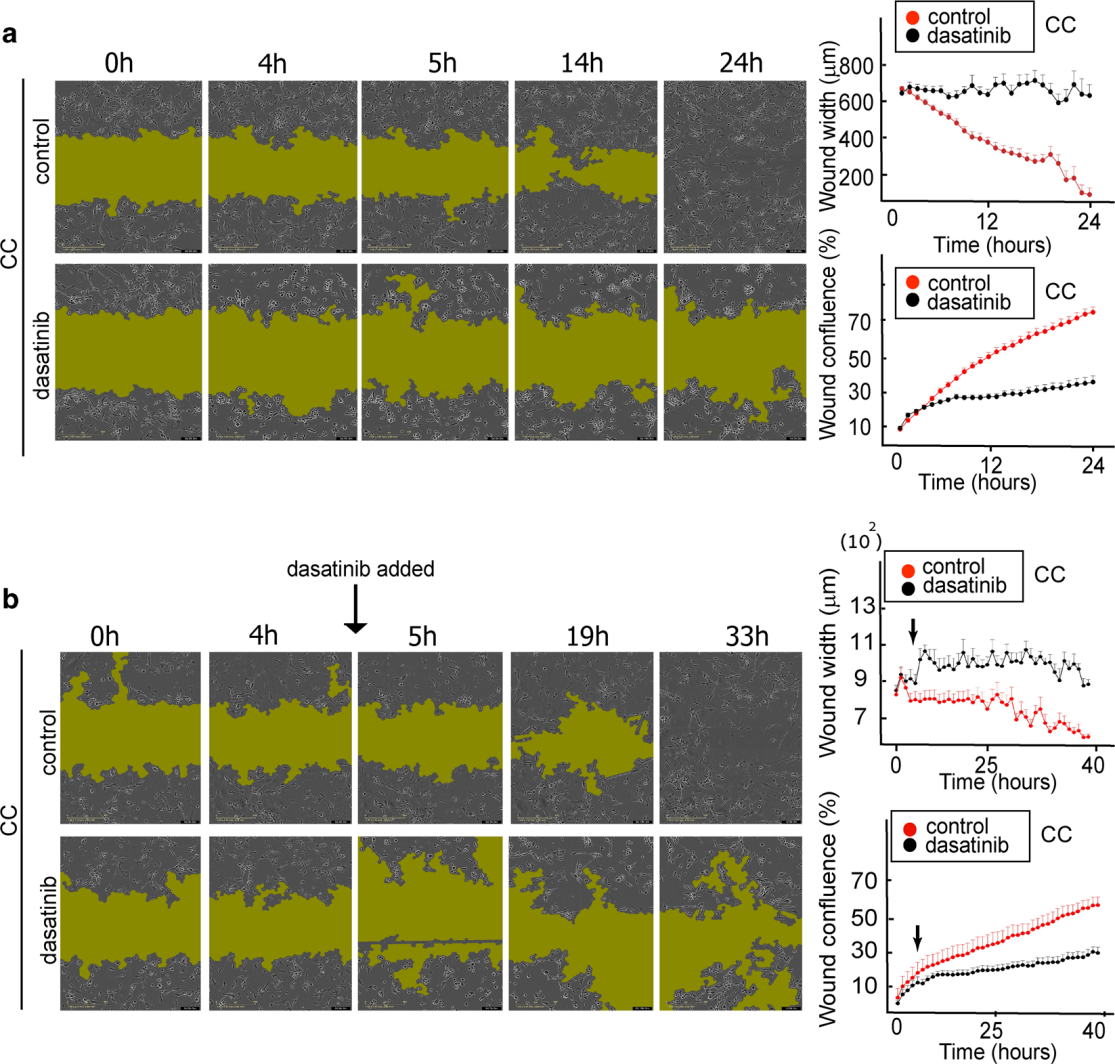
Scratch assay confirms that Src inhibition prevents recolonization of scratch-wound area by EGFRvIII/EGFRwt CC. **a** Left panel; U87EGFRwt cells were co-cultured with U87EGFRvIII cells (CC), in a ratio of 9:1 for 24 h. 100 nM dasatinib was added and movement of the cells into the scratch area was imaged every hour. Representative images for 0, 4, 5, 14, and 24 h are shown for CC and CC + dasatinib. The green shadow indicates the area with very sparse density due to the initial scratch boundary lines. **a** Right panel; wound width and confluence were calculated using the IncuCyte^®^ S3 Software (see also video 3(A and B)). **b** Left panel; U87EGFRwt cells were co-cultured with U87EGFRvIII cells (CC), in a ratio of 9:1 for 40 h. Dasatinib (100 nM) was added between the time points 4 h and 5 h (see black arrow). Movement of the cells into the scratch area was imaged every hour. Representative images for 0, 4, 5, 19, and 33 h are shown for CC and CC + dasatinib. **b** Right panel: quantification of wound width and confluence. *P* value = 0.7 (between the control and treated groups at 4 h), *P* value = 0.005 (between the control and treated groups at 5 h), *P* value = 0.01 (between the treated cells at 4 and 5 h).

We validated our results using a scratch assay, showing that co-cultured cells could not spread into the empty space when they were treated with dasatinib (Fig. 6a). The inhibitor induced directed cell–cell movement towards cellular clusters, thereby inhibiting the recolonization of the scratch area (Fig. 6a, Video 3A,B). Furthermore, dasatinib, which was added ~5 h after the experiment began, reversed the process of the scratch area closure (Fig. 6b). Whereas no significant difference between dasatinib-treated and untreated cells in the process of wound closure was detected up to 4 h, after addition of dasatinib at ~5 h, an expansion of the wound area was detected in the treated cells (Fig. 6b).

This result suggests that Src inhibition not only prevents the tumor cell spreading but also actively reverses the tumor cell infiltration.

Src knockdown using siRNA against Src (Fig. S3A,B) further confirmed the above results. Similar to dasatinib, Src knockdown in U87EGFRwt cells led to the formation of multicellular clusters (Fig. 5h, i) and inhibited the recolonization of the scratch area (Fig. S3C).

To confirm that the effect was specific to Src, U87EGFRvIII/U87EGFRwt co-cultures were treated with an EGFR inhibitor, erlotinib, for 72 h (200 nM and 1000 nM, Fig. S4A). In contrast to dasatinib, erlotinib did not affect the spreading properties of the cells (Fig. 5d, Fig. S4E,F). Furthermore, Figure S4B shows that Src activation was not affected by EGFR inhibition. Additionally, lapatinib, another EGFR inhibitor that has been shown to be escpecially potent in GBM tumors^18^, effectively reduced pEGFR levels (Fig. S4D) when used at non-killing concentrations (Fig. S4A), but failed to induce the formation of multicellular clusters (Fig. S4E,F). Similar results were obtained when the co-cultures were treated with a combination of erlotinib and an anti-EGFR antibody, cetuximab (Fig. S4E,F), or with the anti-mTOR inhibitor, rapamycin (Fig. S5).

### Dasatinib inhibits glioblastoma cell infiltration in 3D models

**Fig. 7:**
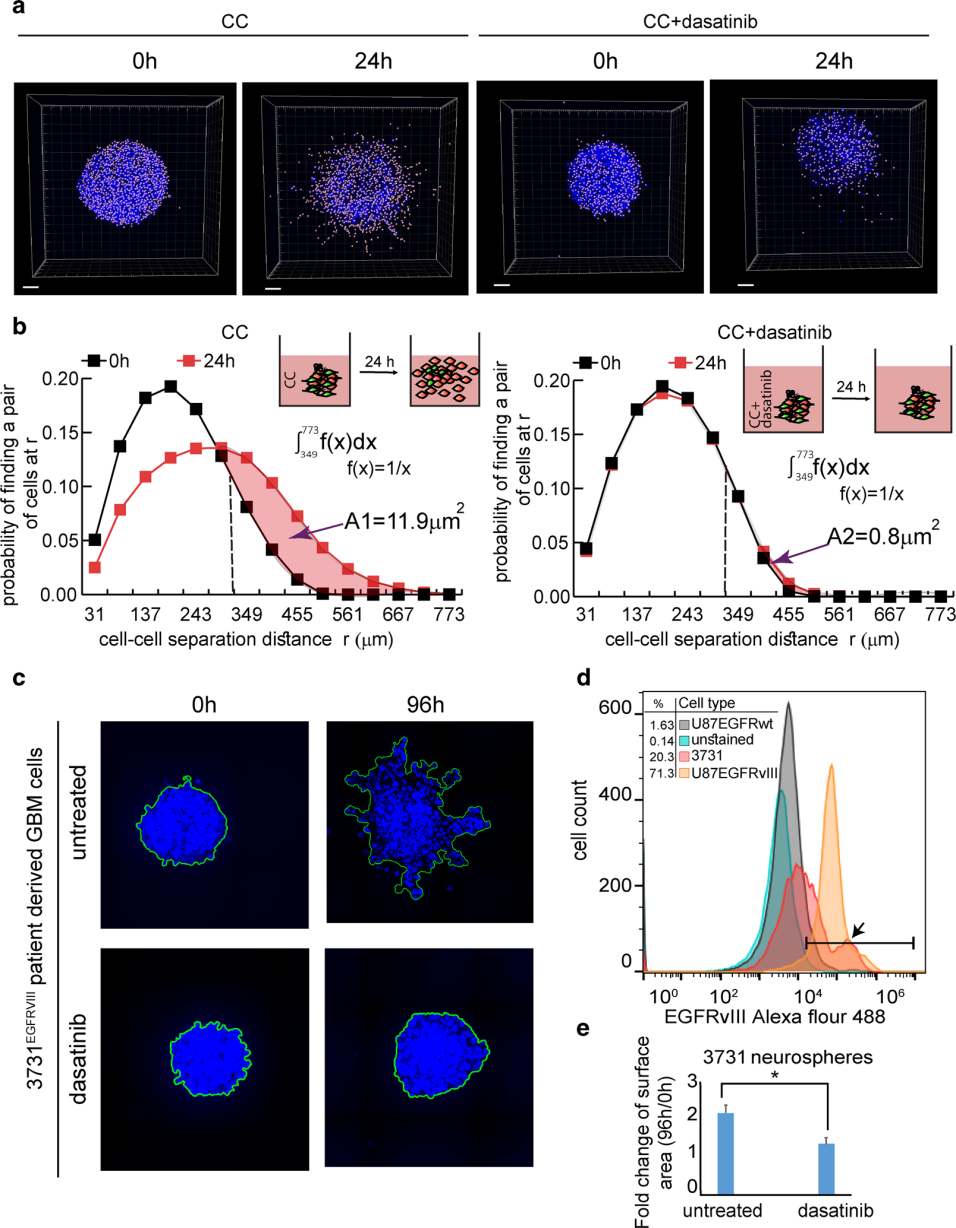
Dasatinib inhibits the infiltrative properties of CC: EGFRwt + EGFRvIII and 3731^EGFRvIII^ NS in 3D. **a**, **b** CC NS were embedded in 40% Matrigel with or without treatment (200 nM dasatinib). **a** Treated and untreated NS were imaged at 0 h and after 24 h, scale bars represent 100 μm. **b** The probability of finding a pair of the cells at *r* (0–800 μm) was calculated at 0 and 24 h as described in Methods. Plots representing the distribution of cell–cell separation distances of treated and untreated CC are shown. The difference between cell–cell separation distance distributions of treated and untreated NS was quantified by calculating the area of shift towards longer cell–cell separations, as shown in the figure. The area indicates the displacement of the distribution between two time points. A large area indicates higher cell spreading. The average area was calculated using four NS for each condition. *P* value = 0.02 between A1 and A2. **c**, **d** The percentage of cells harboring EGFRvIII in the patient-derived 3731 neurospheres was examined using flow cytometry analysis. **d** The histogram indicates the percentage of cells expressing EGFRvIII: U87EGFRwt (1.63%), U87EGFRvIII (71.3%) and 3731 patient-derived GBM cells (20.3%) (indicated with black arrow). **c** 3731 neurospheres were embedded in 40% Matrigel with or without treatment (200 nM dasatinib). Cell spreading was examined for up to 96 h. **e** To quantify neurospheres’ dispersion (which grow as compact structures preventing from the software to resolve clearly single nuclei) 3D neurospheres images were converted similarly to the maximum intensity projection algorithm, namely by projecting 3D structures on 2D surface. The area of each neurosphere between 96 h and 0 h was calculated. *n* = 14 NS for each condition (from two independent experiments). *P value = 0.01.

To confirm that the effect of dasatinib remains similar when GBM neurospheres are cultured in ECM, we performed 3D measurements in matrigel. Figure 7a (see also Fig. S6A) shows that dasatinib significantly reduced the spreading properties of the mixed U87EGFRvIII/ U87EGFRwt (CC) neurospheres. The quantification of cell–cell separation distances following dasatinib treatment is shown in Fig. 7b. Matrigel-embedded CC neurospheres scattered after 24 h as can be seen from the tail of the cell–cell separation distance distribution, which was shifted from the maximum of ~450 μm toward ~700 μm. However, in dasatinib-treated neurospheres the distribution of the cell–cell separation distances remained the same. We quantified the difference between the treated and untreated neurospheres by calculating the area of the shift in the cell–cell separation distributions towards longer cell–cell separations (Fig. 7b). We found that the area of the shift was reduced ~15-fold in response to dasatinib. Importantly, dasatinib did not influence the spreading properties of homogenous U87EGFRwt neurospheres (Fig. S6B).

To determine whether these findings had a potential clinical relevance we utilized patient-derived GBM cells, 3731, expressing EGFRvIII mutation^19^. ~20% of 3731 GBM population harbored EGFRvIII mutation (Fig. 7d). Figure 7c, e demonstrates that following dasatinib treatment the infiltrative properties of the patient-derived 3731 cells were reduced significantly, pointing to the potential success of dasatinib treatment in targeting the GBM infiltration in clinics.

In conclusion, EGFRvIII subpopulations can influence the infiltrative properties of the less aggressive GBM cells through activation of Src signaling. Our findings suggest that non-toxic anti-Src treatment of EGFRvIII-harboring GBM tumors, which was previously found to be ineffective in stopping tumor proliferation, can be effective in inhibiting GBM cell spreading. Tumors which do not express EGFRvIII will not necessarily respond to Src inhibition (Fig. S6B). Thus, Src inhibition might be used as an effective strategy to reduce GBM infiltration of the tumors containing EGFRvIII mutation.

### HGF and IL6 secreted by EGFRvIII cells mediate Src activation in EGFRwt cells

**Fig. 8:**
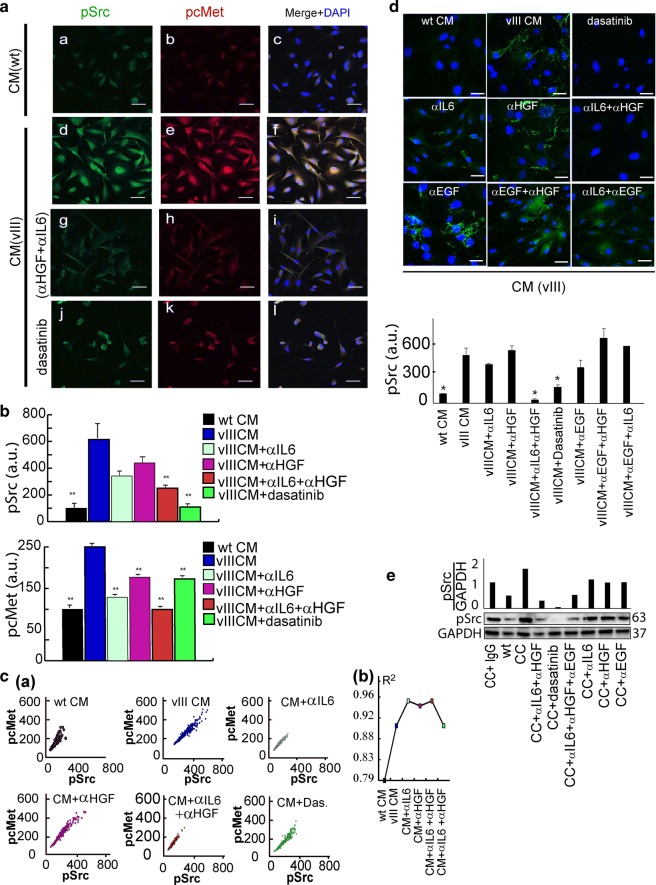
Src activation in EGFRwt cells is mediated via HGF and IL6 secreted from EGFRvIII cells. **a** U87EGFRwt cells were treated for 4 h with: CM of U87EGFRwt CM (WT) (**a**–**c**); CM of U87EGFRvIII (vIII, **d**–**f**); CM of U87EGFRvIII + 0.5 μg/ml (IL6 and HGF) neutralizing antibodies (αIL6 + αHGF) (**g**–**i**); or 200 nM dasatinib (**j**–**l**). Cells were incubated with antibodies against pSrc and pcMet and imaged using secondary antibodies conjugated to Alexa 488 (green) and Cy3 (red) dyes. DNA was stained with dapi. ×40 lens; Scale bar represent 50 μm. **b** Mean intensities of pSrc (*n* = 46–70 cells/condition) and pcMet (*n* = 191–310 cells/condition) were calculated from ~10–13 fields using the NIS-Elements software (Nikon); *P *<* 0.05 and **P *<* 0.005. *P* values representing the significance of the results as compared to U87EGFRvIII CM (vIII). Error bars represent SE**. c** Correlation plots (panel **a**) between pSrc and pcMet were generated for each indicated condition to test co-activation. At least 190 cells were used to generate the plots. R^2^ was calculated for each graph (panel **b**). (**d**, upper panel) U87EGFRwt cells were stimulated with U87EGFRvIII CM, (CM (vIII), excluding the left upper image where the cells were stimulated with U87EGFRwt CM, CM (WT)) and treated with 200 nM dasatinib or different neutralizing antibodies (**α**) (including 0.5 μg/ml αIL6, 0.5 μg/ml αHGF, 0.5 μg/ml αEGF) for 2 h and then fixed for immunofluorescence against pSrc (Alexa 488, green). Cell nuclei were stained with Dapi. Scale bars represent 50 μm. (×40 lens). (**d**, lower panel) Mean intensity of Src activation was quantified for about 180 cells (~13 fields per each condition). *P* values, representing the significance of the difference in pSrc intensity between each treatment and U87EGFRvIII CM, are *P *<* 0.01. **e** U87EGFRwt (WT) cells were co-cultured with 10% U87EGFRvIII, and treated with 0.5 μg/ml αIL-6, 0.5 μg/ml αEGF, 0.5 μg/ml αHGF or 200 nM of dasatinib for 4 h. pSrc levels were examined using Western blot. Quantification of pSrc/GAPDH is shown (upper panel).

To examine the upstream regulators of Src activity, we tested whether the CM of U87EGFRvIII induces activation of one of the central regulators of Src activity—hepatocyte growth factor receptor, cMet^20^. Figure 8a, b (lower panel, blue color) shows that p-cMet was induced when CM of U87EGFRvIII cells was added to U87EGFRwt cells. This result was supported by enhanced coordinated activation of cMet and Src proteins as represented by increased correlation between pSrc and pcMet proteins when U87EGFRwt cells were cultured in CM of U87EGFRvIII cells (Fig. 8c, blue plot and R^2^ plot).

Based on the above results we hypothesized that U87EGFRvIII mediate Src activation in U87EGFRwt cells through HGF ligand^21,22^. To examine whether HGF is involved in the paracrine U87EGFRvIII/ U87EGFRwt cell–cell communication and the increased infiltrative properties of U87EGFRwt cells, we blocked HGF in CM of U87EGFRvIII cells using a neutralizing antibody (**αHGF**).

Blocking HGF alone did not significantly influence Src activity in U87EGFRwt cells (Fig. 8b (magenta color), d, Fig. S7A). Thus, we assumed that an additional factor, secreted by U87EGFRvIII cells, is required to induce Src activity in U87EGFRwt cells. IL6 was recently found to be involved in GBM cell–cell communication^9,10,21^. Indeed, addition of αIL6 to CM which also contained αHGF, significantly reduced Src activity in U87EGFRwt cells (Fig. 8a, b, d). Similar to αHGF, αIL6 alone had less influence on the activity of Src (Fig. 8b, Fig S7A). Thus, at least two secreted factors from CM of U87EGFRvIII cells were required in order to control Src activity in U87EGFRwt cells.

Similar results were obtained in the CC model. Addition of both antibodies was required in order to reduce Src activity in U87EGFRwt cells (Fig. 8d, e). Furthermore, Figure 8d shows that Src was not inhibited when αEGF was combined with either αHGF or αIL6, pointing to the specific effect of the αIL6 + αHGF combination on Src inhibition (Fig. 8d, e). Similarly, adding αEGF to the combination of αIL6 + αHGF did not change the phosphorylation levels of Src in comparison to αIL6 + αHGF alone (Fig. 8e).

**Fig. 9:**
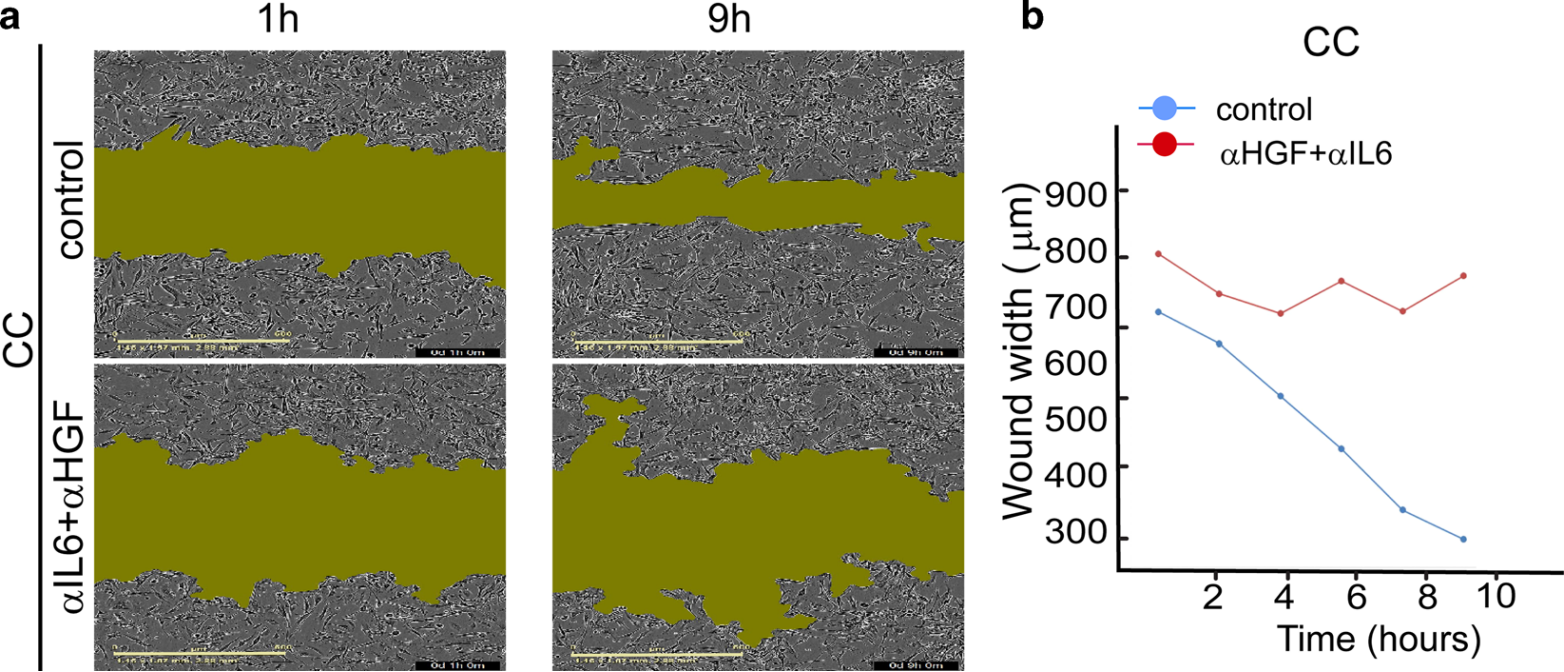
Scratch assay confirms that αHGF and αIL6 inhibit recolonization of the scratch area by EGFRwt/EGFRVIII CC. **a** 0.5 μg/ml αHGF and 0.5 μg/ml αIL6 were added to CC, and movement of the cells into the scratch was imaged for CC or CC + αHGF + αIL6 cells every hour. Representative images for 1 h and 9 h are shown. Green area represents an area unoccupied by the cells (IncuCyte S3 System). **b** The plot describes a change in the width of the “wound” over time. It represents a mean distance between the edges of the wound (in each vertical line resolution) as a function of time. The graph shows one representative experiment out of eight duplicates.

The αHGF + αIL6 combination almost completely stopped the recolonization of the scratch area by the co-cultured cells (Fig. 9). Additionally, U87EGFRwt cell velocity and the length of migration distance were reduced in response to the combination of αHGF and αIL6 (Fig. S7B, C).

These results suggest that U87EGFRvIII-driven paracrine cell–cell communication and induced infiltrative properties of U87EGFRwt cells are mediated through HGF-Src and IL6-Src axes.

## Discussion

Glioblastoma (GBM) is the most aggressive brain tumor and is among the most lethal of all cancers in adults^23^. One of the main reasons for poor patient survival is the diffusive nature of these tumors, resulting in an incomplete resection of the tumors following surgery. Thus, finding an effective strategy to prevent tumor infiltration is an unmet need in clinics.

EGFR and EGFRvIII play critical roles in GBM pathogenesis. Among EGFR mutants found in GBM, EGFRvIII is the most common^8^. Although cells harboring EGFRvIII mutation usually comprise only a small fraction within GBM tumors, this subpopulation plays a crucial role in tumor proliferation^9^.

Here we explore the connection between the paracrine EGFRvIII/EGFRwt crosstalk and the infiltrative nature of GBM tumors. To quantify the infiltrative properties of cancer neurospheres we established a 3D methodology that allows us to calculate cell–cell separation distance distributions in different conditions. We found that aggressive EGFRvIII cells induce the infiltrative characteristics of the less aggressive EGFRwt cells. We show that EGFRvIII cells induce increased cellular velocity and spreading characteristics of EGFRwt cells through soluble factors, IL6 and HGF, and Src activation. Scattered distribution of EGFRvIII/EGFRwt co-culture can be stopped in 2D and 3D matrigel models using Src inhibitor, dasatinib or Src siRNA. Interestingly, dasatinib, used at low, non-toxic concentrations, induces increased cell velocity and directed cell movement of GBM cells toward compacted multicellular structures. We hypothesized that increased cell velocity might occur due to the switch from mesenchymal to amoeboid movement^24,25^, a direction we currently investigate in the lab.

The findings of this study point to the important role of the EGFRvIII/EGFRwt crosstalk in defining scattered GBM architectures and suggest a new direction on how compaction of the infiltrative EGFRvIII GBM tumors can be achieved. Src inhibitors at non-toxic doses, which are not effective in tumor killing^17^, might be assigned a new role in EGFRvIII GBM tumor therapeutics, namely to prevent cell infiltration within the brain. Moreover, we have shown that Src inhibition could actively generate multicellular microstructures thereby reversing tumor cell spreading. Stopping tumor infiltration and/or induction of those microstructures may elevate the probability of the tumor cells to be detected in the brain tissue. Thus, this strategy may have a high potential to improve the outcome of surgical EGFRvIII GBM removal.

In summary, the study reported here provides an additional step toward understanding the infiltrative properties of EGFRvIII GBM tumors. We establish a relationship between the diffusive architectures of tumors harboring EGFRvIII mutations, and communication between tumor subpopulations. We show that the aggressive EGFRvIII subpopulation induces the infiltrative properties of a less aggressive subpopulation via Src activation. Non-toxic Src inhibition could reverse the diffusive properties of the EGFRvIII/EGFRwt population in U87 model and patient-derived GBM cells. Once EGFRvIII mutation can be detected in cerebrospinal fluid through liquid biopsy^11^, non-toxic Src inhibition has a promising potential to enhance the success of EGFRvIII GBM surgeries in the future.

## Materials and methods

### Cell culture

U87EGFRwt and U87EGFRvIII cell lines were engineered from the U87 human glioblastoma cell line^26^ to overexpress wtEGFR (U87EGFRwt) or EGFRvIII (U87EGFRvIII –LacZ), at levels 0.5 × 10^6^ to 1 × 10^6^ receptors per cell^27^, that were found to be consistent with the amplified EGFR levels often found in GBM tumors prepared from patient material^28^. The cell lines were authenticated by the Genomic Center of the Technion Institute (Haifa) and checked on a routine basis for the absence of mycoplasma. Cell lines were routinely maintained as described^9^.

Patient-derived GBM cells: The 3731 patient-derived cell line (PDCL), that expresses EGFRvIII, was cultured as described^19^. Briefly, 3731 PDCL was established by the GlioTex team (Glioblastoma and Experimental Therapeutics) in the Institut du Cerveau et de la Moelle epiniere (ICM) laboratory and maintained in neurosphere growth conditions using DMEM/F12 (Gibco; Life Technologies) culture medium supplemented with 1% penicillin–streptomycin, B27 (Gibco), EGF (20 ng/mL), and FGF (20 ng/mL; Preprotech).

mCherry-labeled U87EGFRwt cells were generated through transduction with a retroviral vector expressing mCherry fluorophore, selected with puromycin (2 μg/ml) and sorted using Fluorescence-Activated Cell Sorter (FACS).

GFP-labeled U87EGFRwt A289V cell line was generated using a retroviral vector expressing EGFR-A289V mutation. The viruses were assembled in HEK293T cells by transfection with 20 μg pHAGE-EGFR-A289V (Addgene 116230), 15 μg pCMVR8.74 (gag-pol-rev-tat) and 5 μg VSV-G (pMD2.G). For generating control cell line, a virus that encodes for Red Fluorescent Protein (RFP) was used. The control virus was assembled using: 20 μg pLV-TRE-RFP 15 μg of pCMV-ΔR8.91 (gag-pol) and 5 μg VSV-G. U87EGFRwt cells were incubated in filtrated viral supernatant (mutation/control) in the presence of 8 μg/ml polybrene (Sigma) for 24 h. After 5–7 d, GFP+/mRFP+ cells were sorted using BD FACS ARIA III cell sorter. Pooled sorted cells were used for the experiments.

For conditioned medium (CM) cells were seeded in our regular DMEM for 24 h, then the medium was replaced with a mild starvation medium (0.5% FCS) and left for 48 h. In certain immunofluorescence experiments, instead of adding CM, we exposed the cells to the medium of the neighbor cells, by lowering the height of the well’s wall of eight wells IBIDI u-slide, in a way that the medium was shared by the cells cultured in different chambers and the chamber-chamber contact was avoided.

In CC experiments 90% of U87EGFRwt and 10% of U87EGFRvIII cells were seeded and incubated in one plate.

### Src knockdown using siRNA

Src siRNA (hs.Ri.SRC.13.1 (IDT, Coralville, Iowa, USA)) was used to knockdown Src in U87EGFRwt cells. Lipofectamine RNAiMax transfection reagent (Invitrogen, New York, USA) was used for siRNA transfection, following IDT recommend protocol. The cells were treated with Src siRNA-lipofectamine or negative control (NC) complexes (0.1–10 nM) in Opti-MEM. 72 h after transfection, Src knockdown was confirmed by Western blotting.

### Antibodies and reagents

The following inhibitors were used: Erlotinib, lapatinib and cetuximab (Erbitux) (for EGFR), dasatinib (for Src) and rapamycin (for mTOR). The inhibitors were diluted in DMSO (equal concentration of DMSO was also used as a control in the appropriate experiments). The following neutralizing antibodies were used: αIL6 (Clone 6708), αHGF (Clone 24612), and αEGF (Clone 10825) and as a control Isotype IgG1 (Clone 11711) (all of them from Bio-Techne).

### Immunoblotting

Immunoblotting was performed as described^29^. The following antibodies were used: anti-EGFR 1:200, anti-mTOR pS(2448) 1:200, anti-ERK pT(202)Y(204) (E-4) 1:200, anti-ERK2 (c-14) 1:200, GAPDH (FL-335) 1:200 (Santa Cruz); pcMet(Tyr1234/1235) 1:1000, anti- EGFR pY(1068) 1:1000,Src (L4A1) 1:1000, and Src pY(416) 1:500,anti-FAK 1:1000, anti-pFAK (Tyr397) 1:1000 (Cell Signaling).

### Flow cytometry

To validate the genomic analysis of 3731 PDLC, the cells were stained with an anti-EGFRvIII primary antibody (L8A4), Santa Cruz, and anti-mouse Alexa Fluor 488 secondary antibody, Jackson immunoResearch. Samples were analyzed using a BD Accuri C6 Flow Cytometer Instrument.

### Survival assay

Methylene blue assays were carried out for 72 h as described^29^.

### Cell movement analysis

U87EGFRwt nuclei were stained with Hoechst 33342, (SC-495790) or labeled with NLS GFP-Luciferase virions (CellLight™ BacMam 2.0, Thermo Fisher). 24 wells plates were coated overnight at 4 °C with 5 μg/ml laminin (LaminStem™ 521, BI #05-753-1F, Biological Industries). After washing, 2 × 10^4^ stained cells were co-seeded with unstained 2 × 10^3^ U87EGFRvIII cells, and tracked for 18 h. In some experiments 1.4 × 10^4^ stained U87EGFRwt cells were cultured in 9 well Ibidi culture plate for 24 h under conditioned medium (CM) of U87EGFRvIII cells (CM; collected following 72 h of incubation with cell in mild starvation(1% FCS)). At each time point multipoint snapshots of ten fields (~150 cell/field) were imaged using 10 × /0.3 Plan Fluor lens mounted on an Eclipse Ti Nikon microscope. Image sequences using Zyla sCMOS (ANDOR) camera, were taken every 20 min. Coordinates of the centers of each labeled nuclei, trajectory, velocity and other parameters were obtained for each cell, in each frame using the Nikon software (NIS-elements).

### Calculations of cell–cell separation distances

~100,000 cells per well were seeded in 6-well plates and incubated at 37 °C for 18 h. Microscopic images were analyzed to obtain the coordinates of the center of each cell. These coordinates were used to calculate the distances between the cells, from cell center to cell center using MATLAB (MathWorks). All cell pairs, up to a separation distance of 400 μm (or as indicated) were measured. The resultant histogram provides a probability for finding a pair of the cells at a certain distance. The obtained probability was divided by a cell–cell distance distributions at 0 h to obtain Radial distribution function (RDF). RDF represents the detected distributions of cell locations with respect to each other^10^.

### Immunofluorescence

Cells were grown on 8 well chambers (Ibidi) coated with laminin, fixed and permeabilized with 100% cold methanol (−20 °C).The slides were incubated with the following primary antibodies p-c-Src (Y416) (9A6) (Santa Cruz) 1:50 and pcMet (Tyr1234/1235) (cell signaling) 1:50 overnight at 4 °C. We used secondary antibodies conjugated to Alexa dyes (647 and 488) or to Cy3- (Jackson Immune Research) 1:100 for 45 min at RT. Mounting was performed with FLUORO-GEL (with DAPI) (Electron microscopy science). Images were acquired using Nikon A1R confocal microscope, with an ×40 lens, 1.3 oil Plan-fluor. The analysis was done using NIS element (Nikon) to obtain the intensity of protein expression per cell.

### Scratch assay

The spreading and migration capabilities of U87EGFRwt cells co-cultured with U87EGFRvIII, were assessed using a scratch-wound assay. 96 wells Nunc plates were coated overnight at 4 °C with laminin as previously indicated. 20 × 10^3^ U87EGFRwt cells were co-seeded with 8 × 10^2^ U87EGFRvIII cells and cultured to nearly confluent cell monolayers. Afterwards a linear wound was generated using the 96-well Wound Maker (IncuCyte®). The plate was incubated in the IncuCyte® S3 Live-Cell Analysis System and tracked for up to 48 h. Images of the scratched areas of each well were obtained every hour. The data were analyzed using IncuCyte® S3 Software 2019A.

### 3D Model

U87EGFRvIII cells labeled with the long term Qtracker705 dye (Molecular Probes), were co-cultured with U87EGFRwt cells in a ratio of 1:9. After 2 weeks of co-culture, neurospheres containing both phenotypes were formed. Nuclei were stained with Hoechst 33342. The neurospheres were seeded in μ-Slide chamber (Ibidi) pre-filled with ice cold liquid Cultrex (basement membrane matrix, mentioned as matrigel in the main text, 40% in DMEM, Trevigen). After 30 min at 37 °C the matrigel has gelified, trapping the neurospheres. Each neurosphere was imaged using a 10×, 0.3 dry EC Plan-Neofluar objective mounted on a ZEISS Confocal LSM710 microscopy. Z-stacks were obtained using 3 μm step size along the axial axis with 80–130 slices in total. During imaging (24 h), cells were maintained at 37 °C and 5% CO2. XYZ coordinates for each cell nucleus were determined using IMARIS 7.2.3. (BITPLANE), and cell–cell separation distances distributions were calculated as described above. All pairs having a distance up to 800 μm were processed as described above.

### In vivo

A mixture of 90% mCherry-U87EGFRwt cells and 10% unlabeled U87EGFRvIII cells was injected via stereotactic surgery into the murine striatum of 7 week old NSG female mice in the right hemisphere, 2 mm lateral and to a depth of 3 mm to the bregma. Each hemisphere was injected with 2 μl of solution, comprised of 150,000 cells/μl resuspended in PBS. The mice were sacrificed after three days, the brains were extracted and visualized using Nikon SMZ18 stereomicroscope and NIS-Elements BR software. All animal experiments were performed in accordance with the guidelines of the Hebrew University committee for the use of animals for research.

### Statistical analysis

Statistical significance was determined by Student’s *t* test (two tails, two samples equal variance); *P* values of ≤0.05 were considered statistically significant. All data represent the mean ± S.E. (standard error of the means). If not indicated otherwise, the experiments were performed at least three times.

## Supplementary information

VIDEO1A

VIDEO1B

VIDEO2A

VIDEO2B

VIDEO3A

VIDEO3B

SUPPLEMENTAL MATERIAL
