# Supplementary material for: Dissecting the role of crosstalk between glioblastoma subpopulations in tumor cell spreading
Source: Oncogenesis. 2020 Feb 5;9(2):11. doi: 10.1038/s41389-020-0199-y (PMC7002777; doi:10.1038/s41389-020-0199-y)
Supplement: Supplementary file 7 — SUPPLEMENTAL MATERIAL [file 41389_2020_199_MOESM7_ESM.docx]

S**upplementary file**

**Dissecting the Role of Cross-talk Between Glioblastoma Subpopulations in Tumor Cell Spreading**

# Maria R. Jubran^#1^, Ariel M. Rubinstein^#1^, [Irina Cojocari](https://openscholar.huji.ac.il/natalykracvhenkoblashalab/people/irina-cojocari?ref_tid=7413)^#1^, Ibukun Adesoji Adejumobi^1^, Maxim Mogilevsky^2^, Sama Tibi^1^, Ronit V. Sionov^1^, Maïté Verreault^3^,[Ahmed Idbaih](https://www.ncbi.nlm.nih.gov/pubmed/?term=Idbaih%20A%5BAuthor%5D&cauthor=true&cauthor_uid=31706351)^3^, Rotem Karni^2^ and Nataly Kravchenko-Balasha^1^*

^1^Department for Bio-medical Research, Faculty of Dental Medicine, Hebrew University of Jerusalem, Jerusalem 91120, Israel

^2^Department of Biochemistry and Molecular Biology, Institute for Medical Research Israel-Canada, Hebrew University-Hadassah Medical School, Jerusalem 9112001, Israel

^3^ Sorbonne Université, Inserm, CNRS, UMR S 1127, Institut du Cerveau et de la Moelle épinière, ICM, AP-HP, Hôpitaux Universitaires La Pitié Salpêtrière, Service de Neurologie 2-Mazarin, F-75013, Paris, France

**^#^** Equal contribution

**Figure S1**

**
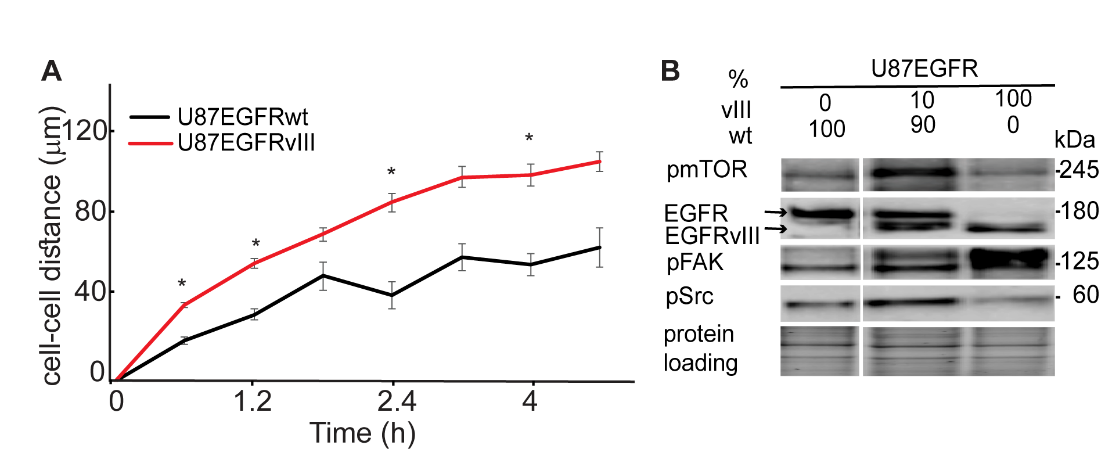
**

**Fig S1.** **U87EGFRvIII daughter cells reach larger cell-cell separation distances than U87EGFRwt daughter cells after cell division**. (**A**) Using live-cell imaging, cell-cell separation distances between each pair of daughter cells was measured as described in video 1 legend. (**P* < 0.05) (error bars, represent Standard Error (SE)). (**B**) **pSrc induction is detected following the** **addition of 10% of U87EGFRVIII to CC.** pSrc416, pFAK and pmTOR were induced when 90% of U87EGFRwt cells were co-cultured with 10% of U87EGFRvIII for 24h.

**Figure S2**

**
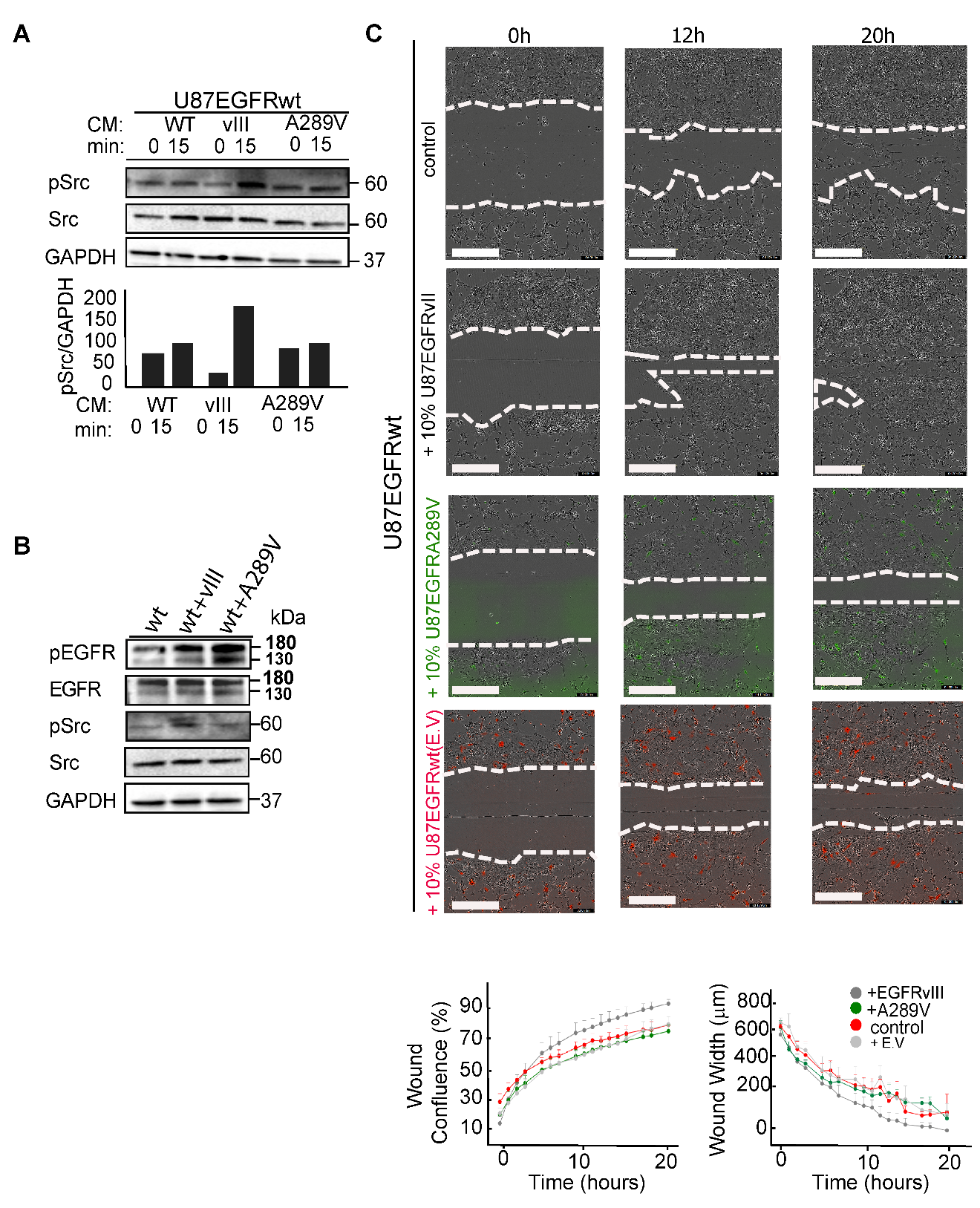
Fig S2.**  **10% of** **U87EGFRA289V cells do not induce neither Src phosphorylation in U87EGFRwt cells nor mimic the EGFRvIII migratory effect when co-culture with 90% of U87EGFRwt cells. (A-C)** In these experiments U87EGFRwt cells with activating EGFRA289V mutation (see methods) were used**.** This mutation was shown previously to induce the invasive phenotype when expressed in 100% of GBM cells. (**A**) U87EGFRwt cells were incubated in either U87EGFRvIII CM (vIII), U87EGFRA289V CM or their own CM (WT) medium. Western blot assay was performed to examine the effect of U87EGFRvIII CM (vIII)/ U87EGFRA289V CM on U87EGFRwt cells after 15 mins. Bottom panel: quantification of pSrc levels normalized to GAPDH. **(B)** pSrc416 was induced when 90% of U87EGFRwt cells were co-cultured with 10% of U87EGFRvIII for 24h. However pSrc levels remained essentially unchanged when 90% of U87EGFRwt cells were co-cultured with 10% of U87EGFRA289V cells. **(C)** Scratch assay: Upper panel: U87EGFRwt cells were co-cultured with either U87EGFRvIII cells (CC), U87EGFRA289V GFP+ cells, or U87EGFRwt RFP+ cells (Empty Vector (E.V.)) for 24 hrs in ratio of 9:1. Movement of the cells into the scratch area was imaged every hour. Representative images for 0, 12, and 20 h are shown. The recolonization rate of the scratch area remained essentially the same when either 10% of U87EGFRA289V GFP+ or U87EGFRwt RFP+ were added to 90% of U87EGFRwt cells. The dashed lines indicate the remained wound width. Scale bars represent 400μm. (**C**) Lower panel: wound width and confluence were calculated by the IncuCyte® S3 Software.

**Figure S3**

**
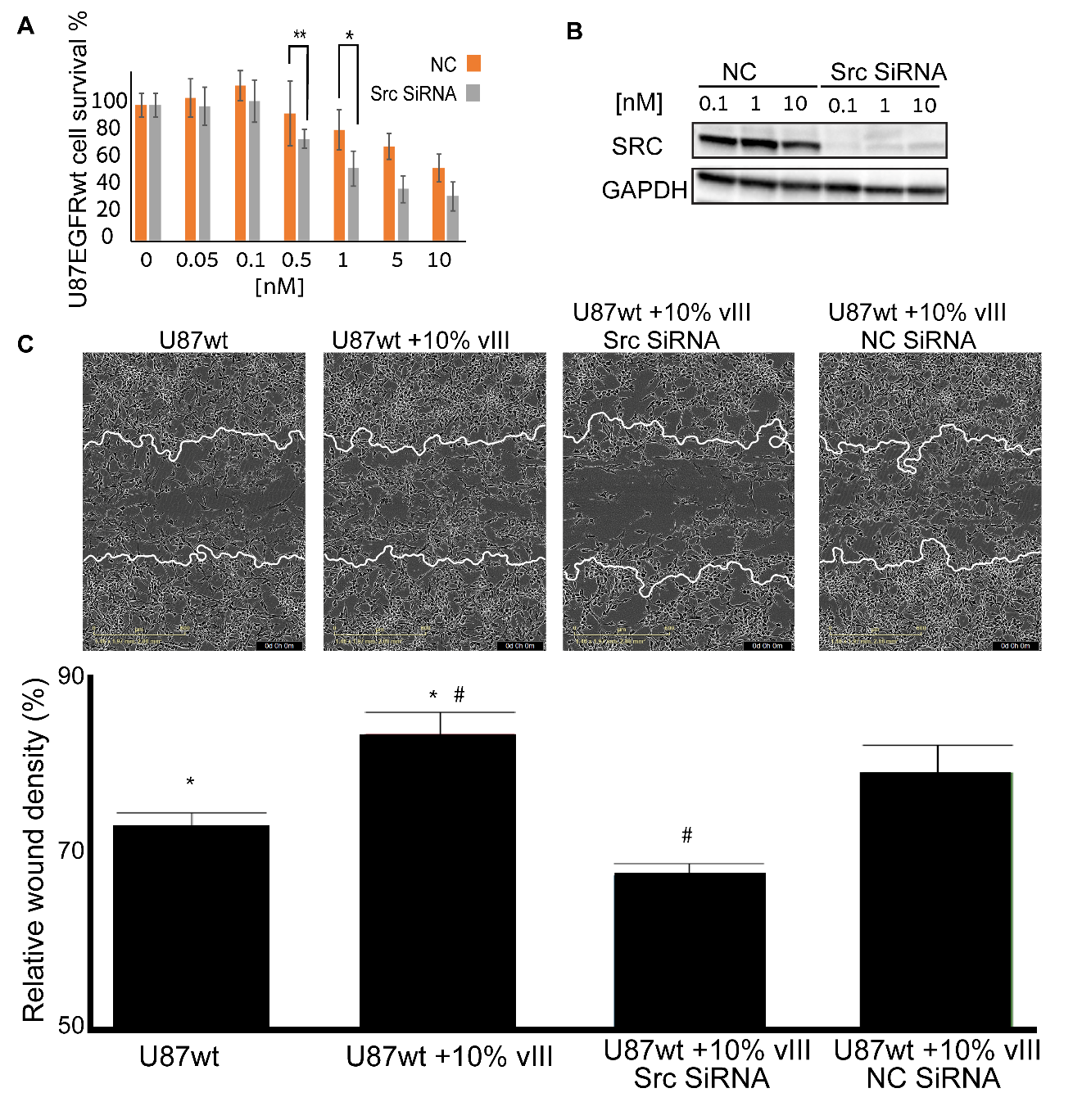
Fig S3.** **U87EGFRwt cells transfected with Src siRNA demonstrate reduced ability to close the scratch area in CC. (A)** U87EGFRwt cells were treated for 72 h with 0-10nM Src SiRNA or with Negative control (NC) to determine an effective working concentration that allow at least 80% viability. Bars represent % of viable cells as assessed by Methylene blue assay. **P* < 0.05; ***P* = 0.1 (SE are shown). (**B**) 0.1nM Src siRNA was enough to knockdown Src protein. For further analysis 0.5nM were chosen due to a clear phenotypic effect. (**C**) U87EGFRwt cells were transfected with 0.5 nM of either Src SiRNA or Negative control SiRNA. After 24h, 25x10^3^  cells co-cultured with U87EGFRvIII cells (CC), in ratio of 9:1. Scratch area recolonization was recorded and quantified. Upper panel: Representative images of each treatment (20h after the wound was generated) are shown. The white line indicates the original scratch boundary. Lower panel: wound density was calculated by the IncuCyte® S3 Software, **P* < 0.02; #*P* = 0.001.

**Figure S4**

**
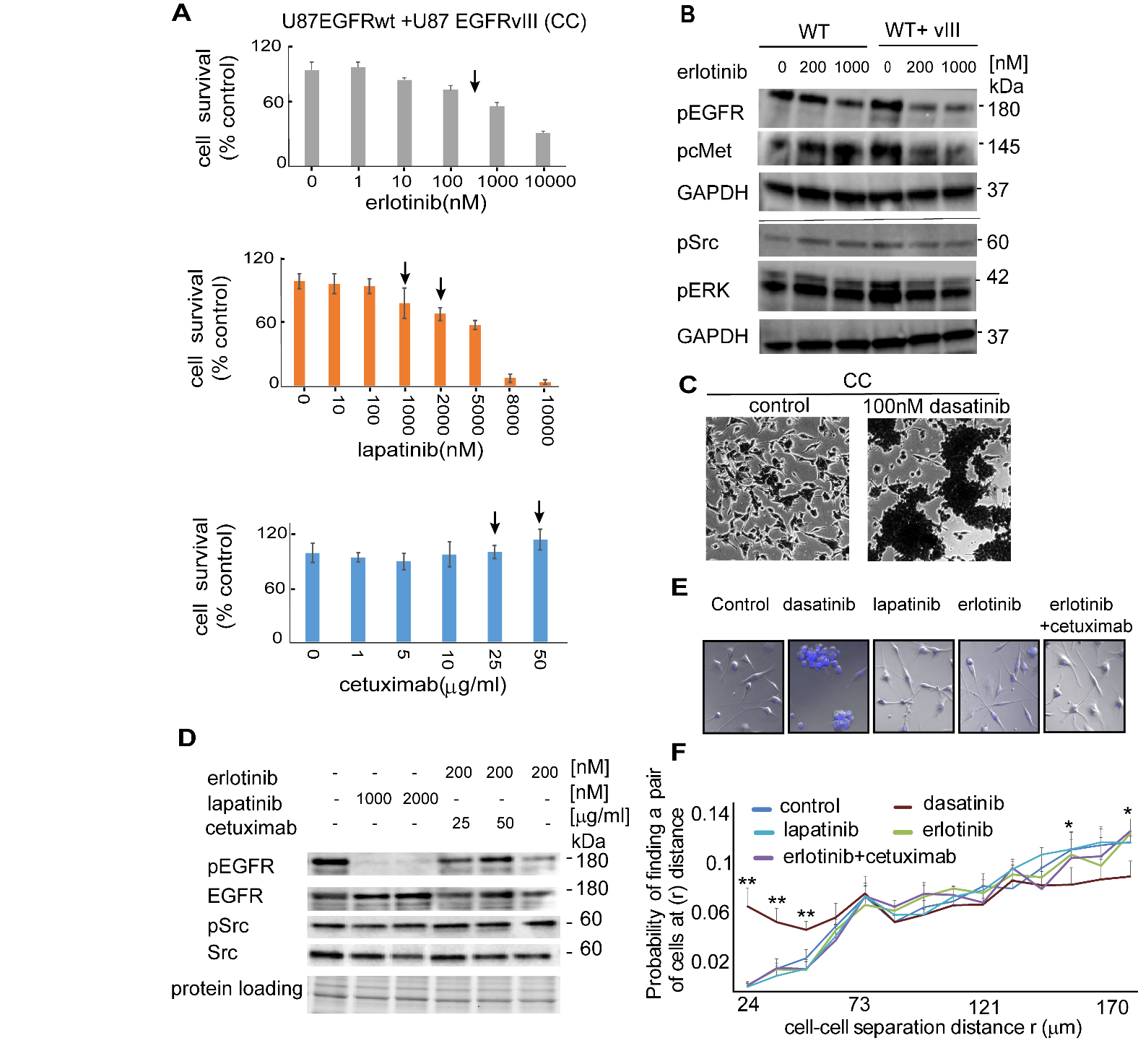
**

**Fig S4.** **Inhibition of EGFR does not affect Src activation. (A)** U87EGFRwt cells were co-cultured with 10% U87EGFRvIII cells and treated for 72h with erlotinib (0-10μM), lapatinib (0-10μM) and of cetuximab (0-50 μg/ml) to determine the optimal concentration for each inhibitor that allow survival of ~ 80% cells (black arrow indicate the selected concentrations). Error bars represent SE**. (B)** Western blot analysis shows that 200nM Erlotinib inhibits pEGFR, pcMet, pERK but fails to inhibit Src (Y416) activation. **(C)** Dasatinib induces cellular microstructures: CC cells were treated with either DMSO or dasatinib (100nM) for 72h. Fixed cells were imaged after methylene blue staining. **(D)** Inhibition of pEGFR after 72h by lapatinib (1-2 μM), erlotinib alone (200nM) or in combination with cetuximab (25 μg/ml ). The drugs reduce pEGFR to various degrees, (lapatinib was highly effective), but fail to inhibit Src (Y416) activation in U87EGFRwt cells co-cultured with 10% U87EGFRvIII cells. WB assays were repeated in two independent experiments**. (E,F)** Unlike dasatinib, EGFR inhibitors don’t induce cellular aggregation: CC cells were treated with either DMSO, dasatinib (100nM), lapatinib (1uM), erlotinib (200nM) or erlotinib+ cetuximab (25μg/ml) for 24h. Hoechst-labeled cell nuclei were imaged using live-cell imaging chambers (Eclipse Ti-E, Nikon inverted microscope Scale bars, 100μm, 10x lens). Results from **(E)** are plotted in (**F**)**.** The curves in the graph (**F**) represent distributions of the cell-cell separation distances in the cultures treated with dasatinib/ lapatinib/ erlotinib with or without cetuximab or of the untreated cultures. **P* < 0.01, ***P* < 0.0001 were found between the dasatinib and the others treatments.

**Figure S5**

**
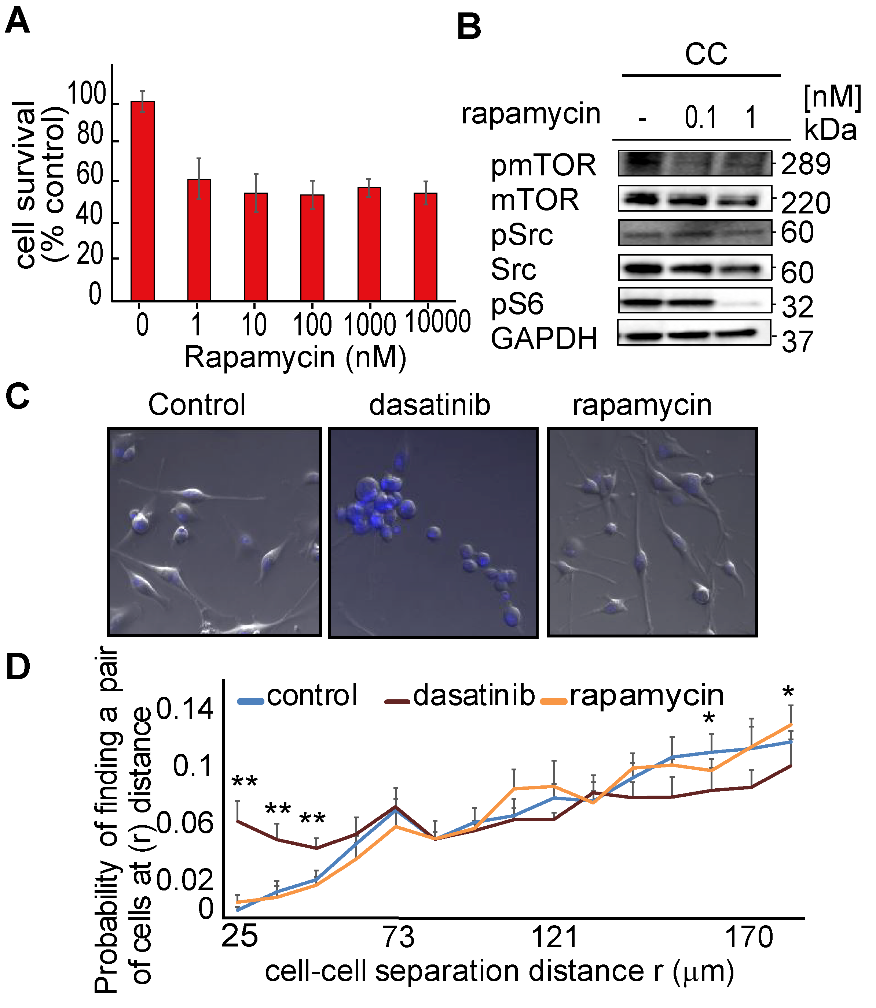
**

**Fig S5.** **Inhibition of mTOR does not affect Src activation. (A)** U87EGFRwt cells were co-cultured with 10% U87EGFRvIII cells and treated with rapamycin (pMTOR inhibitor, 0-10μM) for 72 h. 0.1 nM and 1 nM were selected for further experiments. Error bars represent SE**. (B)** CC was treated with rapamycin (0.1, 1 nM) for 72h. Following mTOR inhibition no reduction in Src (Y416) activation was noticed. WB results were repeated in two independent experiments**. (C,D)** Unlike dasatinib, mTOR inhibitor did not induce cellular aggregation: CC cells were treated with either DMSO, dasatinib (100nM) or rapamycin (1nM) for 24h. Hoechst-labeled cell nuclei were imaged using live-cell imaging chambers (Eclipse Ti-E, Nikon inverted microscope Scale bars, 100μm, 10x lens). (**D**) represents quantification of (**C**). The curves in the plot represent the distributions of cell-cell separation distances in the cultures treated with dasatinib/ rapamycin or in untreated cultures. **P* < 0.01, ***P* < 0.001 represent the difference between control and dasatinib and control and rapamycin treatments correspondingly.

**Figure S6**

**
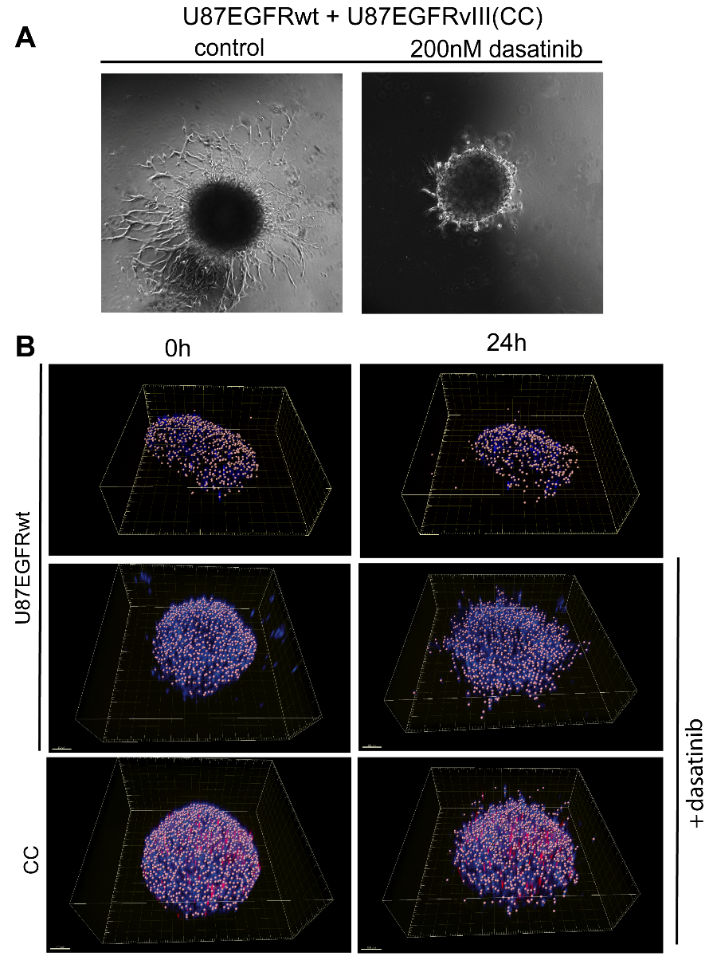
**

**Fig S6.** **Dasatinib inhibits the infiltrative properties of CC** **neurospheres (NS) but not of U87EGFRwt NS.** (**A**) CC (90% of wt and 10% of U87EGFRvIII) GBM neurospheres, generated during 2 week incubation, were embedded in 40% Matrigel. Cells were treated for 24h and fixed in 4% PFA. Hoechst-labeled nuclei were imaged after 24h (scale bars represent 100μm). (**B**) GBM NS were generated as mentioned above, containing either 100 % of U87EGFRwt cells (upper and mid panels) or CC (90% of U87EGFRwt cells and 10% of pre labeled U87EGFRvIII cells with Qtracker705, displayed in red, lower panel). 200nM of dasatinib blocked the dispersion of the CC but did not effect 100% U87EGFRwt NS. U87EGFRwt NS did not spread significantly after 24h (upper panel). Hoechst-labeled cell nuclei were imaged at 0h (left panel) and 24h (right panels) using confocal microscopy.

**Figure S7**

**
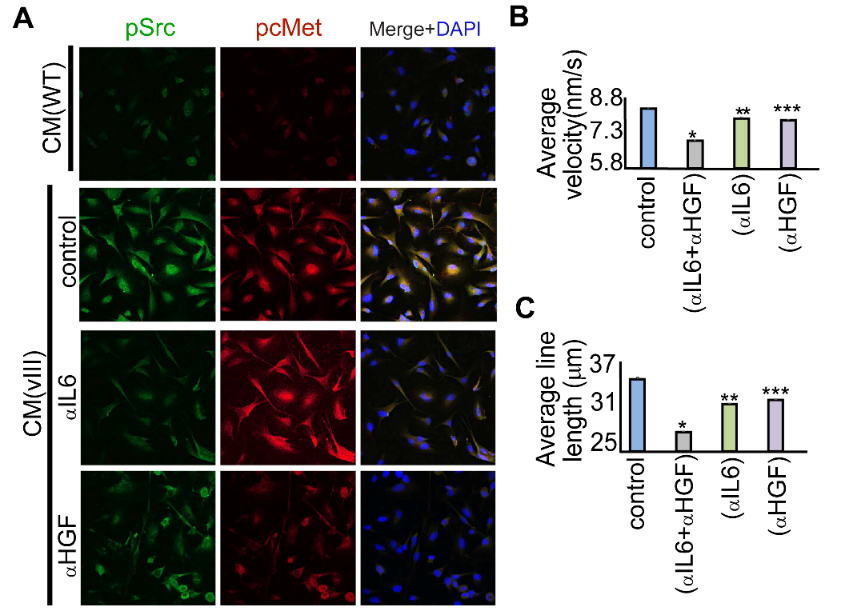
**

**Fig S7.** **Src activation is mediated via HGF and IL6.** **(A)** U87EGFRwt cells were treated for 4h with U87EGFRvIII CM or with neutralizing antibodies (α) (0.5μg/ml αIL6 or 0.5 μg/ml α HGF). Cells were incubated with antibodies against pSrc and pcMet and imaged using secondary antibodies conjugated to Alexa 488 (green) and Cy3 (red) dyes. DNA was stained with dapi. 40x lens; Scale bar represent 50μm. Images are representative of at least three independent experiments (**B and C**) U87EGFR wt cells were co-cultured with U87EGFRvIII and treated with αIL6 , αHGF or a combination of αIL6 + αHGF (0.5 μg/ml for all reagents). Hoechst-labeled cell nuclei were imaged for 18h using live-cell imaging chambers; 10x lens (Eclipse Ti-E, Nikon inverted microscope). Average velocity (**B**) and line length (**C**) were calculated using the NIS-Elements software (NIKON). (**B**) **P < 0.0032*; ***P* = 0.36; ****P* = 0.19. (**C**) **P* < 0.02; ***P* = 0.34; ***P* = 0.51. The presented data was collected from at least 3000 cells of total 8 movies. Data are representative of at least three independent experiments.

**Video Legends:**

**Video 1: U87EGFRvIII cells display a stronger repulsion forces between the just dividing cells than U87EGFRwt cells**. U87EGFRwt (**video 1A**) and U87EGFRvIII (**video 1B**) nuclei were labeled with NLS GFP-Luciferase virions to facilitate the tracking analysis. Thereafter the cells were cultured on laminin pre-coated 24 wells plates and tracked for 18 h. For both videos multipoint snapshots of ten fields were taken every 20 min. X, Y coordinates of the center of each labeled nuclei, trajectory and velocity were obtained for each cell, using the Nikon software (NIS-elements). Plot in Fig S1A represents this video.

**Video 2: Dasatinib leads to cellular clustering**

U87EGFRwt cells were co-cultured with U87 EGFRvIII cells (CC) in ratio of 9:1, in laminin pre-coated 6 wells plates **(video 2A)**. CC was treated with 100nM of Dasatinib **(video 2B)**. In both videos ten fields (at least 150 cell/field) were tracked for 18 h. Multipoint snapshots of the fields were taken every 20 min. X, Y coordinates of the center of each labeled nuclei, trajectory and velocity were obtained for each cell (NIS-elements). The video supports the results shown in the Figures 5D-G.

**Video 3: Dasatinib prevents cell spreading and actively induces cellular clusters**

U87EGFRwt cells were co-cultured with U87EGFRvIII cells (CC) in ratio of 9:1, for 24 h with or without 100nM Dasatinib. After the generation of a wound in the cell culture, the plates were incubated in the IncuCyte® S3 Live-Cell Analysis System and tracked for 24 h. Images of the scratched areas in each well were taken every hour. Movement of the cells into the wound was tracked for untreated CC cells (**video 3A**) and CC cells treated with Dasatinib **(video 3B**). Scale bar represent 600μm.The video supports Figure 6A.
